# Effects of machine learning errors on human decision-making: manipulations of model accuracy, error types, and error importance

**DOI:** 10.1186/s41235-024-00586-2

**Published:** 2024-08-26

**Authors:** Laura E. Matzen, Zoe N. Gastelum, Breannan C. Howell, Kristin M. Divis, Mallory C. Stites

**Affiliations:** Sandia National Laboratories, Mail Stop 1327, P.O. Box 5800, Albuquerque, NM 87185-1327 USA

**Keywords:** Visual search, Machine learning errors, Human-system interaction

## Abstract

This study addressed the cognitive impacts of providing correct and incorrect machine learning (ML) outputs in support of an object detection task. The study consisted of five experiments that manipulated the accuracy and importance of mock ML outputs. In each of the experiments, participants were given the T and L task with T-shaped targets and L-shaped distractors. They were tasked with categorizing each image as target present or target absent. In Experiment 1, they performed this task without the aid of ML outputs. In Experiments 2–5, they were shown images with bounding boxes, representing the output of an ML model. The outputs could be correct (hits and correct rejections), or they could be erroneous (false alarms and misses). Experiment 2 manipulated the overall accuracy of these mock ML outputs. Experiment 3 manipulated the proportion of different types of errors. Experiments 4 and 5 manipulated the importance of specific types of stimuli or model errors, as well as the framing of the task in terms of human or model performance. These experiments showed that model misses were consistently harder for participants to detect than model false alarms. In general, as the model’s performance increased, human performance increased as well, but in many cases the participants were more likely to overlook model errors when the model had high accuracy overall. Warning participants to be on the lookout for specific types of model errors had very little impact on their performance. Overall, our results emphasize the importance of considering human cognition when determining what level of model performance and types of model errors are acceptable for a given task.

**Supplementary Information:**

The online version contains supplementary material available at 10.1186/s41235-024-00586-2.

## Significance Statement

As machine learning (ML) algorithms are adopted into more and more contexts, it is important to consider how these tools impact their end users’ workflow and decision-making. In particular, it is crucial to consider how ML errors can impact human performance. Past research on automated systems shows that automation failures can be catastrophic. Yet much of the current research on ML research assumes that the algorithms will make very few errors. No algorithm will ever perform perfectly under all circumstances, but there has been relatively little research on the impact of ML errors on human decision-making. In high-consequence domains where ML stands to greatly benefit users, we must also address the potential impact of ML errors, especially if those errors are rare and difficult for users to notice. In this paper, we report a series of experiments that used tightly-controlled mock ML outputs to test the impact of ML errors on human performance in a target detection task. We found that models with low overall accuracy could improve human performance, but participants were less likely to notice ML errors when the errors were rare. Missed targets were harder for participants to detect than model false alarms, and higher proportions of misses among the ML errors led to lower human performance. Finally, warning participants to watch for missed targets did not improve their performance, although warning them about model errors in general had some benefit. Our findings have implications for future research and development of ML algorithms that can provide better support to human decision-making.

**Supplementary Information:**

The online version contains supplementary material available at 10.1186/s41235-024-00586-2.

## Introduction

The use of machine learning (ML) to aid human decision-making is growing in popularity across a wide variety of domains, including medical diagnosis, system failure detection, threat detection, and automated vehicle operation (Du et al., 2019; Goddard et al., 2014; Körber et al., 2018). Human users interact with ML-based systems in a multitude of ways, and these systems are designed to function as a human–machine team. Even when the ML algorithm provides a suggested decision, the human user is still the final decision maker in the process. For several types of tasks, such as classifying the topics of research abstracts (Goh et al., 2020), recognizing traffic signs (Ciresan, Meier, Masci, & Schmidhuber, 2011), geolocating photographs (Weyand, Kostrikov, & Philbin, 2016), and navigating high-speed video games (Fuchs, Song, Kaufmann, Scaramuzza & Deurr, 2021), ML model performance now exceeds human performance. However, because no algorithm will perform perfectly, it is critical to understand how easily users can notice and overcome errors made by a model. Models can make a variety of errors, such as false alarms (falsely indicating that a target of interest is present when it is not) and misses (failing to detect a target that is present).

Related work has tested how system-level characteristics impact human trust in systems that include automation (Meyer, 2004; Rice, 2009). Previous research has suggested that false-alarm prone systems may elicit different patterns of trust in the system, and thus different patterns of human performance, relative to miss-prone systems (Meyer & Lee, 2013, Rice & McCarley, 2011; Dixon & Wickens, 2006; Dixon et al., 2007). While both error types reduce compliance with and reliance on an automation, false-alarm prone systems lead to significantly less compliance, suggesting that false alarms may be more salient to users than misses (Rice, 2009, Rice and McClary, 2011).

The impact of different types of system errors may also depend on the application area in which the model is being used. Depending on the operational context in which the model is being deployed, missing a target of interest could have devastating consequences, whereas false alarms may cost time or resources but be within the operational limitations and expectations of users. Airport security and medical image analysis are examples of cases where false alarms are tolerated in order to avoid missing critical anomalies (cf. Biggs et al., 2018). In other contexts, such as severe weather forecasts, misses and false alarms are both problematic. Failure to predict a severe event such as a tornado can put lives at risk, but too many false alarms might decrease the public’s trust in the forecasts and make them less likely to heed severe weather warnings in the future (cf. Ripberger et al., 2015).

Prior research on computer aided detection (CAD) has found that viewers can become over-dependent on CAD cues when looking for targets in images, such as cancer in a mammogram. Accurate CAD cues can lead to improved human performance, but if the system does not correctly mark a target, the viewer is very likely to miss that target (Drew et al., 2012, 2020; Kunar, 2022; Kunar et al., 2017a, 2017b; Kunar et al., 2017a, 2017b). While increased miss rates are a pressing concern for CAD systems, research on other types of automated systems has identified situations in which false alarms are more detrimental to human performance than misses. For example, when participants were given a tracking task and a simultaneous automation-aided system monitoring task, they had poorer task performance when the automation was prone to false alarms than when it was prone to misses (Dixon et al., 2007). With the miss-prone system, the participants monitored the raw data, relying less on the automation. In the false alarm-prone system, the frequent false alarms slowed the participants down because they had to evaluate every alert to determine whether or not it was real. This hurt their overall task performance and also produced more errors in the cases where the automated alerts were accurate. Similar issues with false alarms have been observed in contexts such as physical security. When users must evaluate every alert produced by an automated system, having too many false alarms can lead to an unacceptable decline in human-system performance (Bandlow et al., 2017).

Most efforts to develop ML algorithms to assist humans in these scenarios focus on maximizing the overall performance of the algorithm, yet no model will achieve perfect performance at all times. There are no agreed-upon thresholds for how “good” a model needs to be before it can be deployed for use in a decision support context. Similarly, very little research has been done to assess how model errors impact the human users of the model. Rather than focusing on algorithm performance alone, we argue that it is crucial to consider the human side of the human-algorithm team in order to determine whether the performance of an algorithm is acceptable for a given task. As discussed above, we have reason to believe that certain types of ML errors, such as missed targets, will be more difficult for humans to detect when reviewing a model’s outputs. We also have reason to believe that it will be harder for users to detect ML errors when those errors are rare. It is well-established that humans struggle to detect rare targets (Horowitz, 2017; Hout et al., 2015; Peltier & Becker, 2016; Rich et al., 2008; Wolfe et al., 2005). When a person is using a high-performing algorithm, that algorithm’s errors may be similarly difficult to detect.

In this paper, we present five experiments that systematically varied the accuracy of a mock ML algorithm. The participants were given the T and L task, a classic visual search task in which the target is a T with a perfectly centered crossbar and the distractors are L-like shapes with an offset crossbar (Wolfe et al., 1989). In Experiment 1, the participants performed the task without algorithmic assistance. They viewed a series of images and categorized each one as “target present” or “target absent.” In Experiments 2–5, the participants received assistance from a mock ML model that placed a bounding box around each target that it detected. They were told that the model’s outputs were not accurate 100% of the time, but that the outputs were intended to assist them with the target detection task. On most trials, the mock ML outputs were correct. If there was a T present in the image, there was a bounding box around it. If no T was present, there was no bounding box. However, some trials contained incorrect ML outputs. These could be false alarms (FAs), where the bounding box marked a non-target L, or misses where a T was present in the image but was not marked. There were also combined FA/miss trials, where the image contained a target, but the bounding box marked an L elsewhere in the image.

It is important to emphasize that this study used *simulated* machine learning outputs. This allowed us to manipulate whether the model provided a correct or incorrect response at the stimulus level, and to control the exact proportion of correct and incorrect responses at the experiment level. The drawback of using outputs from an actual ML model is that the stimuli for which the model made an incorrect decision could be systematically different from those it answered correctly, producing confounds in the stimuli. Creating simulated model outputs allowed us to isolate the impacts of incorrect ML suggestions on human performance outside of the bounds of any single ML model implementation.

Across the experiments in this study, we manipulated the accuracy of the mock ML outputs and the types of errors that they produced. In Experiment 2, we varied the accuracy of the ML outputs from 50 to 95% correct in order to test the impact of increasing model accuracy on the participants’ ability to detect increasingly rare model errors. We predicted that participant performance would generally improve when using more accurate models. However, given that people have a difficult time detecting rare targets, we predicted people would have a hard time detecting rare model errors as well. That is, we predicted that when a model had high performance overall, people would likely demonstrate higher model compliance (or willingness to go along with its suggestions), making them less likely to notice incorrect model suggestions. A secondary question was whether participants’ performance in response to different types of model errors (i.e., FAs and misses) would be differentially impacted by the model’s overall accuracy.

In Experiment 3, we manipulated the types of errors produced by the model, using a subset of the error rates from Experiment 2. In some cases, most or all of the errors were misses, while in others most or all of the errors were false alarms. We predicted that participants would perform better when the model produced more FAs than misses, since the FAs were much easier to detect.

In Experiment 4, we manipulated the relative importance of different types of items. One group of participants was told that they should do their best to categorize all of the images correctly (similar to the prior experiments). Another group was told that the target present items were most important, so they should make sure that those items were categorized correctly, regardless of the accuracy of the ML outputs. A third group was told that the target absent items were most important. We predicted that the emphasis on target present stimuli would draw participants’ attention to the items that contained bounding boxes, improving their performance on trials where the ML produced a false alarm. Similarly, we predicted that the emphasis on target absent stimuli would draw participants’ attention to the items that did not contain bounding boxes, potentially improving their performance on the trials where the model missed a target. Experiment 4 was intended to reflect the real-world scenario where certain types of model errors are more consequential than others.

Experiments 2–4 all focused on human performance: participants were instructed to categorize the images correctly even if the model outputs were not accurate. Experiment 5 changed the focus to model’s performance. Like in Experiment 4, one type of item (target present or target absent) was deemed to be more important than the other. However, in Experiment 5, the participants were warned to be on the lookout for cases where the model categorized those items incorrectly. When target present items were emphasized, the participants were instructed to be on the lookout for cases where the model missed a perfect T. We expected that these instructions would improve participants’ performance on the trials where the model missed the target. When the target absent items were emphasized, the participants were instructed to be on the lookout for cases where the model produced a false alarm. We expected this to improve the participants’ performance on the model FA trials.

Our experiments found that even very poorly-performing ML models can benefit users by making the task easier when the model outputs were correct. However, as predicted, when the model accuracy was very high, the participants became less likely to notice the model errors. Their performance was much poorer overall for cases where the model missed a target than for cases where the model produced a false alarm. In this task, identifying a false alarm was much easier than finding a missed target. We found that changing the instructions to emphasize one type of item over another had relatively little impact on the participants’ ability to identify model FAs. However, emphasizing human performance and correct categorization of target absent trials led to improved accuracy on the trials where the model missed a target. The results of these studies highlight the importance of considering human cognition when determining what ML accuracy rates and error types are acceptable in any given context.

## Experiment methods

The five experiments in this study used the same set of materials and the same data collection procedures. In this section, we describe the methods that were consistent across experiments.

### Ethics

All of the experiments in this project were reviewed and approved by the Human Studies Board at Sandia National Laboratories.

### Materials

**Fig. 1:**
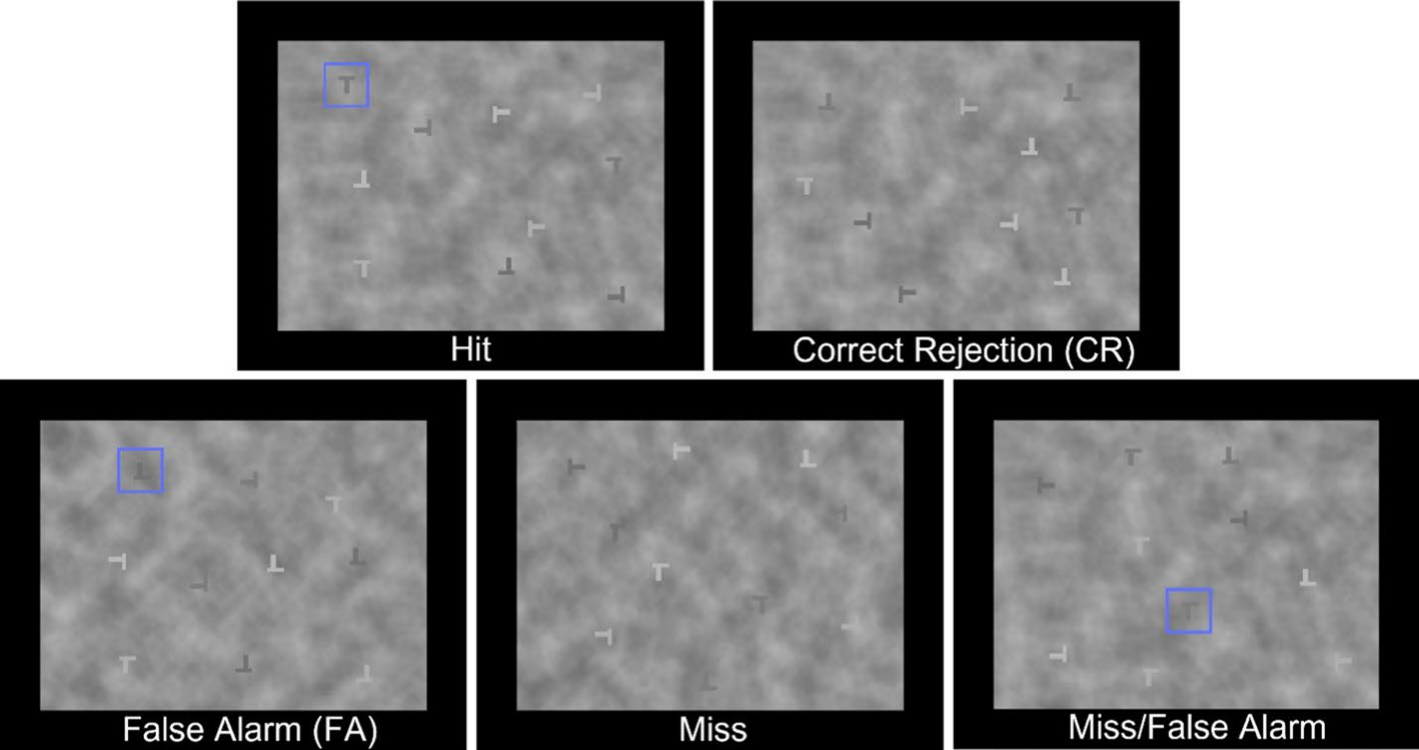
Examples of the stimuli used in all five experiments. The stimuli used in Experiment 1 did not contain bounding boxes. The stimuli in Experiments 2–5 had bounding boxes marking the targets in Target Present images and no bounding box in Target Absent images. However, the bounding boxes were not always correct, as seen in the model false alarm, miss and miss/false alarm conditions

All five experiments used the same set of materials for the T and L task. The stimuli were grayscale images with a cloudy background and a black border, as shown in Fig. 1. The items placed on the background were Ts with perfectly centered crossbars (targets) and Ls, which were similar to the Ts but with off-center crossbars (distractors). The Ts and Ls could appear in four different orientations, with the crossbar pointing up, down, left or right, and in four different shades of gray: two light and two dark. Each stimulus had a total of 10 letters, five of which were one of the lighter shades of gray and five of which were one of the darker shades of gray. The targets appeared equally often in each of the four quadrants of the image (top left, top right, bottom left, and bottom right).

The stimuli were developed in matched sets so that targets and distractors of the same color appeared in the same location across conditions. Some of the letters were easier to discern than others because of the contrast between the color of the letter and the color of the background at a given location. The matched stimuli ensured that there were no systematic biases that could make the targets more difficult to discern in one condition than in another. The distractors that were not matched to a specific target location were moved around so that the appearance of targets and distractors in the same locations across different stimuli was not obvious to the participants.

In Experiment 1, participants completed the T and L task without assistance from the mock ML algorithm. This allowed us to establish a baseline level of human performance for the task. In Experiments 2–5, bounding boxes were added to the images to represent ML outputs. The bounding boxes were blue squares that were placed around one of the letters in the image, as shown in Fig. 1. Superimposed bounding boxes are a common representation of ML outputs in target detection tasks and applications that are intended to support human visual search performance. In a prior study, we found that bounding boxes led to better performance in a similar T and L task than other representations of ML outputs (Matzen et al., 2021a). Across stimuli, the placement of the bounding boxes was counterbalanced such that they appeared equally often in every quadrant of the image.

There were five model output conditions. In two of the conditions (hit and correct rejection) the model’s output was correct. In the hit condition, a target appeared in the image and a bounding box was correctly placed around the target. In the correct rejection (CR) condition, the image did not contain a target and no bounding box appeared. In the other three conditions (false alarm, miss, and false alarm/miss), the model’s output was incorrect. In the false alarm (FA) condition, there was no target in the image, but a bounding box appeared, placed around one of the distractor Ls. In the miss condition, a target appeared in the image, but no bounding box appeared. Finally, in the FA/miss condition, a target and a bounding box both appeared in the image, but the bounding box was incorrectly placed around one of the distractor Ls rather than the target T.

Each experimental list contained 120 stimuli where 72 of the images (60%) contained a target and 48 (40%) did not. Experiments 2–5 varied how often the mock ML outputs correctly identified images with and without targets. Tables showing the number of stimuli in each condition for each experiment are available in the Supplemental Materials.

### Procedure

Participants completed the experiments via Amazon Mechanical Turk. We requested that they use a laptop or desktop computer to complete the experiment so that they would not have to scroll to see the images in their entirety. After completing the consent form, the participants read the instructions for the task: “In this experiment, you will see images that contain T-shaped items on a foggy background. Most of the items have offset crossbars so that they look a little like an L, but some are perfect Ts with a centered crossbar. The perfect Ts and the offset Ts can appear in any orientation (pointing up, down, left, or right). Your goal is to determine whether or not there is a perfect T in each image as quickly as possible. It doesn’t matter if the T is upright, upside down, or rotated to the left or right. Any of those variants counts as a T, so long as the crossbar is perfectly centered.” In Experiment 1, these instructions were followed by example images with and without targets.

In Experiments 2–5, the participants saw additional instructions explaining the bounding boxes. Those instructions read: “As you complete this task, you will also see information produced by a machine learning algorithm. The algorithm places a blue box around the shape that it thinks is a perfect T. If the algorithm thinks there is NOT a perfect T in the image, it does not place a box on the image. The indicator from the machine learning algorithm may not be accurate 100% of the time, but it is intended to help you search the images faster.” The participants were then shown examples of a target present image with a correctly placed bounding box (a hit), a target absent trial with no bounding box (a CR), and a target present image with an incorrectly placed bounding box (an FA/miss). They were reminded of the appropriate response for each of those examples. Finally, they were told: “Remember that the machine-generated information **may not be accurate 100% of the time,** but it is intended to help you. Your job is to correctly indicate whether a perfect T is present in the image, regardless of the presence or accuracy of the bounding box indicator.”

**Fig. 2:**
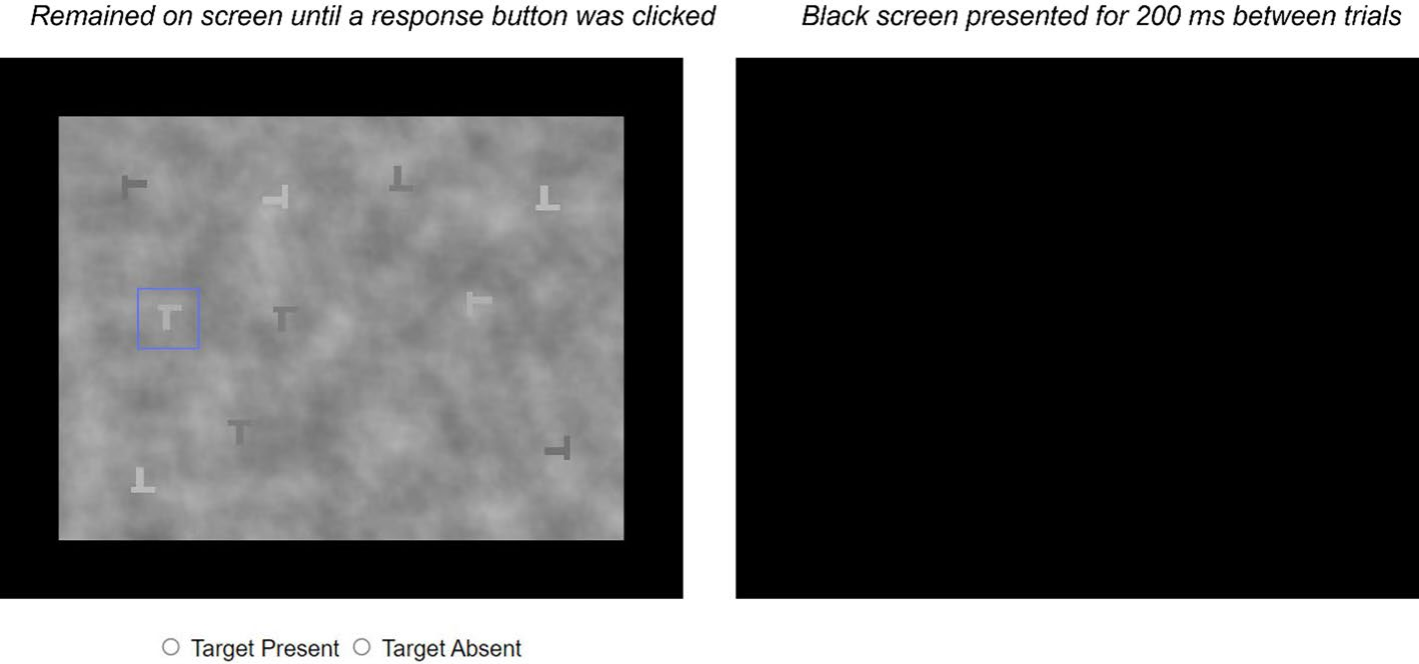
Each trial consisted of a visual search stimulus that remained on the screen until one of the radio buttons was clicked, followed by a black screen. This example shows an FA/miss trial where the bounding box incorrectly marks a distractor rather than the target. Note that the trials in Experiment 1 had the same structure, but none of the images contained a bounding box

After reading the instructions, the participants clicked a button to begin the task. All of the images were scaled to be 550 pixels wide. There were radio buttons labeled “Target Present” and “Target Absent” below the image and participants clicked on the buttons to select their response. When the participants clicked on the buttons, a black rectangle that matched the size of the stimuli was presented for 200 ms and then the next trial appeared. The participants did not receive feedback about their responses. An example of the trial structure is shown in Fig. 2.

Participants saw 120 stimuli plus 10 catch trials which were presented in a random order. The catch trials simply told participants which of the response buttons to click. These were included to make sure that the participants were actually looking at the images while completing the task. At the end of the experiment, participants were asked what kind of device they had used to complete the experiment (desktop, laptop, tablet, or mobile device). They were also asked to estimate how many of the trials contained a target (Experiment 1) or how many trials had a correct model output (Experiments 2–5). They selected a button corresponding to their estimate, and their response options were 20%, 30%, 40%, 50%, 60%, 70%, 80%, 90% or 100%.

### Participants

Participants were recruited via Amazon Mechanical Turk and were paid $2.00 for their participation. They were required to have completed at least 1000 prior HITs with an approval rate greater than 95% and they were required to be located in the USA. The participants could not participate in more than one of the experiments in this study.

In Experiment 1, there were numerous participants (21 out of 57) who responded randomly rather than following the task instructions. These participants tended to alternate between the two response buttons or to click the same response button for many trials in a row. Their patterns of responses made it clear that they were not following the task instructions or searching the images for the targets. In order to remove the participants who responded randomly, we adopted two exclusion criteria. Participants were excluded if they responded incorrectly to three or more of the ten catch trials. Participants were also excluded if their accuracy on the visual search trials was less than 60% correct. We selected this threshold because 60% of the images contained targets, so if participants could achieve 60% accuracy if they clicked the “Target Present” button on every trial (as some participants did). These exclusion criteria successfully removed the participants who appeared to be responding randomly, so the same criteria were used for all of the experiments.

Due to the number of poorly performing participants in Experiment 1, we required the participants in Experiment 2 to have the “master” qualification from Mechanical Turk. This qualification is given to Turkers who complete many tasks and consistently produce high quality data. We found that the master participants were less likely to submit random responses. Ideally, we would have continued to use the master participants for Experiments 3–5. However, we found that the pool of masters was not large enough to support the high number of participants required for those experiments. Thus, for Experiments 3–5 we adopted the strategy of allowing participants who did not have the master qualification, but collecting data from more participants than our desired sample size. This enabled us to have roughly the same number of participants in each experimental list even after dropping the poor performers. Note that the participants’ performance in Experiments 3–5 was considerably higher than it was in Experiment 1 due to the addition of the mock ML outputs, which made the task much easier overall.

Table 1 provides an overview of all five experiments, including the variables manipulated in each experiment, the number of conditions, the total number of participants, and the number of participants who were removed from the analysis based on our exclusion criteria. Summaries of the results of the experiments are shown in Tables 3 and 4. Table 3 shows the participants’ average overall accuracy in each experimental condition, while Table 4 shows their accuracy for the subset of trials on which the ML output was incorrect.

**Table 1:** Overview of the five experiments

Expt	Key manipulation	# of conditions	Total # participants	Participants excluded (< 60% accuracy and/or missed > 3 catch trials)	Participants remaining in analysis
1	Baseline—no “ML” assistance	1	58	21	37
2	Error rate	6	216	8	208
3	Error type	18	669	82	587
4	Item importance (emphasis on human performance)	9	347	44	303
5	Error importance (emphasis on detecting model errors)	9	345	45	300

## Experiment 1: human performance without model assistance

As outlined above, Experiment 1 required the participants to complete the T and L task without ML assistance. The participants saw stimuli that did not have bounding boxes and were simply asked to categorize each image as “Target Present” or “Target Absent.”

### Results

The first step in the analysis process was the removal of trials with extremely long or short response times (RTs). Since the data collection took place online, participants sometimes stepped away from the task, leading to RTs of 10 min or more on individual trials. At the opposite extreme, participants occasionally clicked one of the response buttons too quickly, before evaluating the stimulus. To eliminate these outliers, we removed all trials where the participant’s RT was more than three standard deviations (SD) from their mean RT. In addition, we removed all trials where the response time was less than 500 ms or more than 60 s. We made the assumption that RTs less than 500 ms were likely to be an accidental button press rather than a realistic evaluation of the stimulus. Similarly, we assumed that the participant had been distracted by something else if the trial lasted for more than 60 s (the mean RT across all trials and all participants was approximately 5 s). A total of 61 out of 4,440 trials were dropped, with no more than five trials dropped from any one participant. Of those, 43 had an RT more than 3 SD from the participant’s mean RT, seven had an RT less than 500 ms, and 11 had an RT longer than 60 s.

**Fig. 3:**
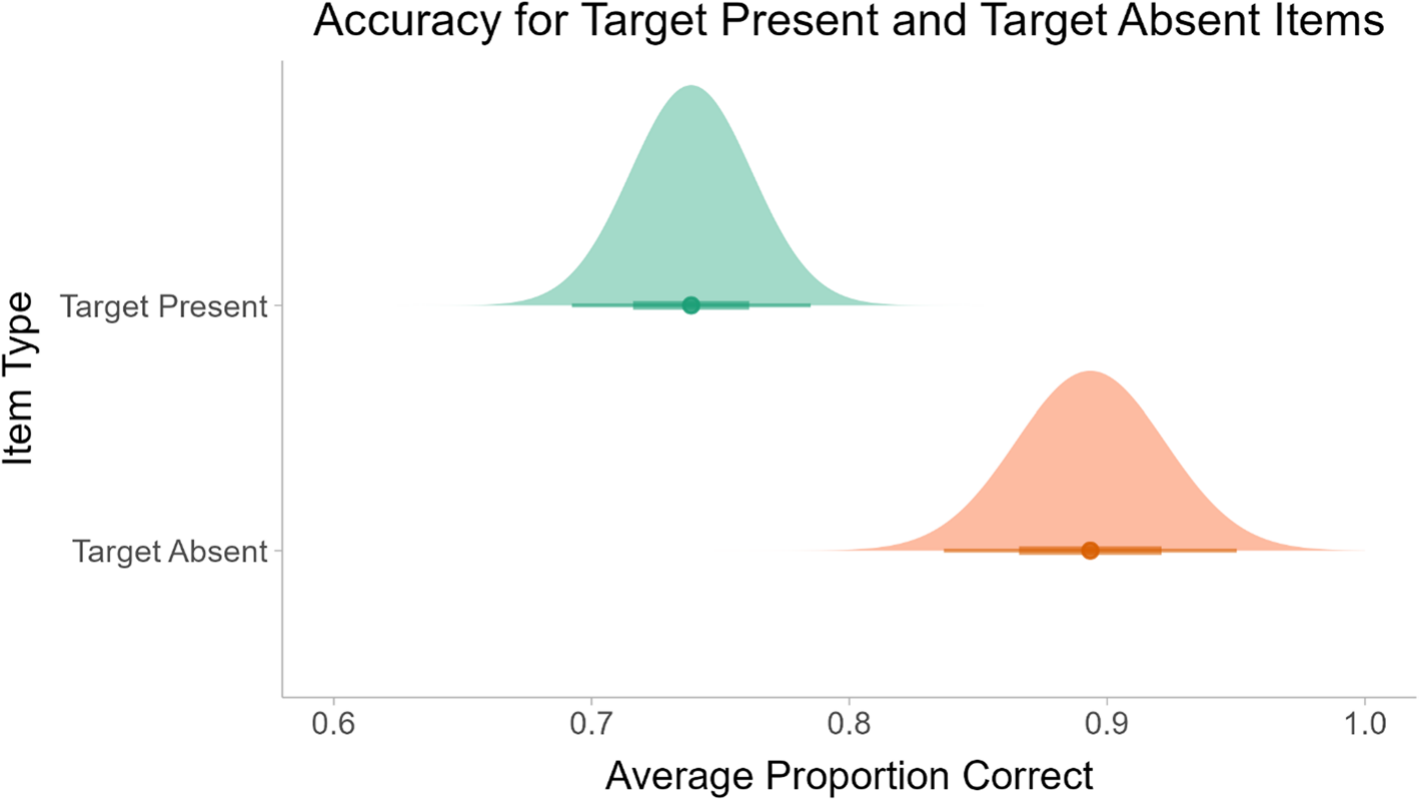
Mean proportion correct for the target present and target absent stimuli. Circles and lines denote the median, 66% and 95% confidence intervals

The participants’ overall mean accuracy was 80.2% correct (SD = 9.5%). The analysis compared the participants’ performance for target present and target absent stimuli. A paired t-test showed that the participants had significantly higher accuracy for the target absent items than for the target present items (*t*(36) = 3.68, *p* < 0.001). These results are shown in Fig. 3.

**Fig. 4:**
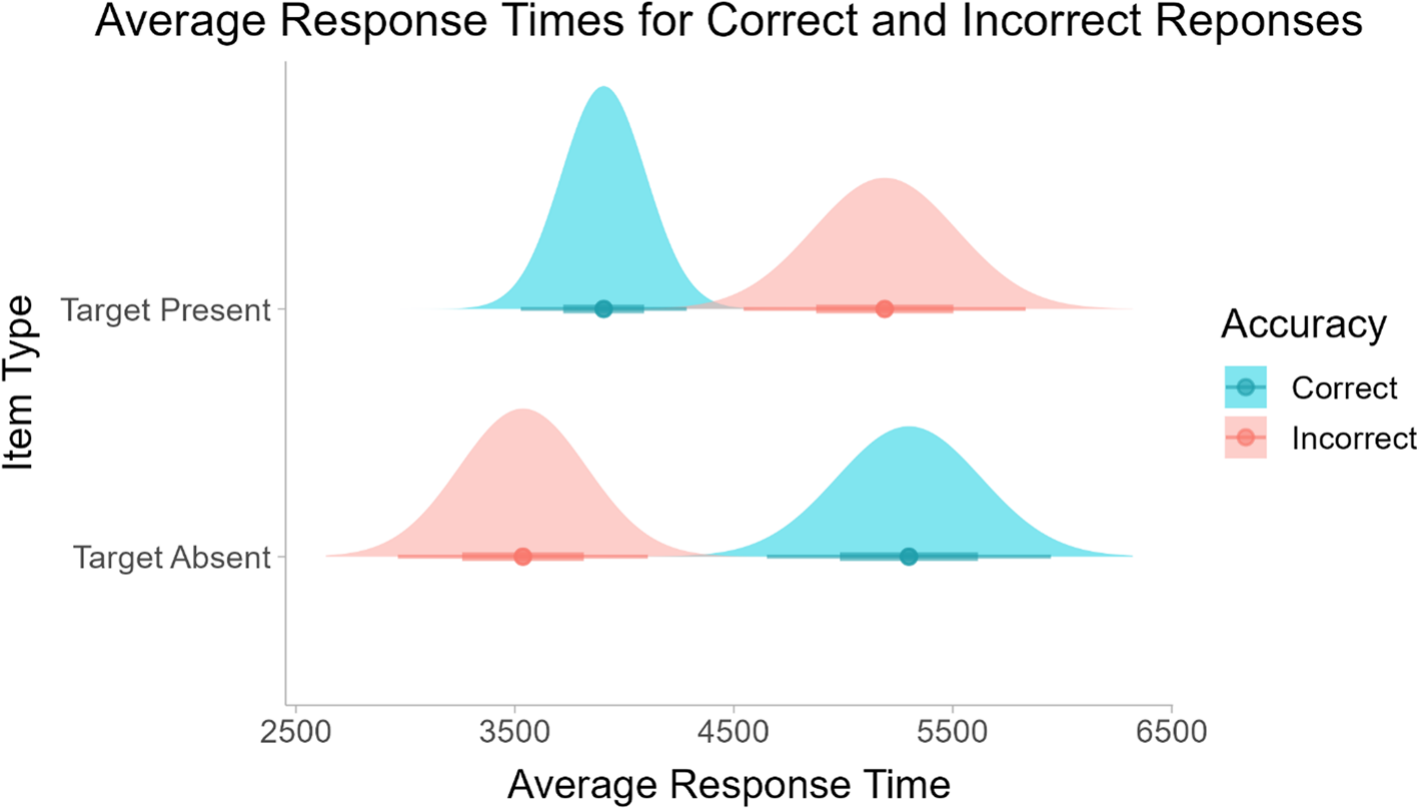
Mean response times for correct and incorrect responses to both stimulus types

Figure 4 shows the average RTs for correct and incorrect responses to target present and target absent stimuli. A two-way repeated measures ANOVA was used to analyze the RTs for the participants’ correct and incorrect responses to the two types of stimuli. The ANOVA found a significant interaction between stimulus type (target present or target absent) and response accuracy (*F*(1,23) = 49.86, *p* < 0.001). Pairwise t-tests with Bonferroni correction showed that when a target was present, participants were significantly faster when they found the target than when they did not (*p* < 0.01). For images that did not contain a target, the opposite was true. In that case, participants had significantly longer RTs when they correctly answered “Target Absent” (*p* < 0.001). Similarly, when participants got the answer correct, they responded significantly faster when the image contained a target than when it did not (*p* < 0.001). When the participants answered incorrectly, their responses were significantly slower when the image contained a target than when it did not (*p* < 0.001).

The participants’ performance on the T and L task can also be analyzed using signal detection theory to calculate their sensitivity in distinguishing targets from non-targets (*d’*) and their decision criterion (*c*). Analyses of *d’* and *c* for all of the experiments can be found in the Supplemental Materials.

### Discussion

The results of Experiment 1 were consistent with prior studies of the T and L task. The participants found about 74% of the targets, on average, but missed a sizable proportion. When the participants correctly identified a target, they had significantly shorter RTs than when they missed it or when there was no target to find. Upon finding a target, the participants could end their search. However, if there was no target or if the participants could not find it, they spent more time searching the images before responding.

The subsequent experiments added mock ML outputs in the form of bounding boxes to the experimental stimuli. Experiments 2–5 tested the impact of those outputs, particularly incorrect outputs, on human performance for this target detection task.

## Experiment 2: manipulating model error rate

In Experiment 2, bounding boxes were added to the T and L images, as described in the Experiment Methods section. The participants were told that the bounding boxes were produced by a machine learning model and that they were intended to assist them with finding the targets in the images. The accuracy of the “model outputs” was manipulated across conditions to test the impact of model accuracy on human performance. There were six experimental conditions in which the model outputs were correct 50%, 60%, 70%, 80%, 90%, or 95% of the time. There were three different types of model errors (misses, FAs, and FA/misses), which occurred equally often within each experimental list. For example, in the 80% accuracy condition, there were eight misses, eight FAs, and eight FA/misses. A table listing the number of items in each condition is provided in the Supplemental Materials.

Our hypothesis in Experiment 2 was that the participants’ overall performance would improve as the model accuracy increased, providing them more assistance with the task. However, we also hypothesized that participants would be less likely to notice the model errors when they were rare.

### Data cleaning

After excluding participants who missed three or more catch trials (one participant) or whose overall accuracy was less than 60% (seven participants), there were 216 participants remaining, with 34–36 participants per error rate condition. As in Experiment 1, the trials with unusually long or short RTs were removed from the analysis. However, we used a slightly different data cleaning procedure for the experiments that included mock ML outputs. In Experiment 1, the participants had to conduct a visual search on every trial, but in Experiments 2–5, many trials had correctly-placed bounding boxes that eliminated the need for visual search (i.e., all model hits). This was the most common trial type in all of the experimental lists and the participants could respond very quickly to those trials, leading to lower mean RTs overall. However, when the bounding box was absent or placed incorrectly, the participants still needed to search the images to verify whether or not there was a target present. Their RTs for this subset of trials were sometimes longer than the mean + 3 SD cutoff that we used in Experiment 1. We wanted to exclude trials where participants were distracted or stepped away from the task, but not those where they were completing the task appropriately by conducting a careful visual search. Therefore, we decided to retain all trials with an RT less than 15 s, even if those trials were more than 3 SD from that participant’s mean RT. The 15 s cutoff was selected based on the results of Experiment 1, where the overall mean RT (5.02 s) plus one SD (9.9 s) was just under 15 s. Based on the patterns observed in Experiment 1, it seemed that search times under 15 s were very likely to reflect a legitimate effort to search the image rather than instances where the participants’ attention was diverted from the task.

Our data cleaning procedure for Experiment 2 (and all subsequent experiments) was as follows:

1. Calculate the mean RT and SD for each participant.
2. Drop all trials with RTs less than 500 ms or greater than 60 s (as in Experiment 1).
3. Drop all trials with RTs longer than the participant’s mean + 3 SD, but *only* if those RTs were longer than 15 s.

For Experiment 2, this process led to the exclusion of 117 out of 24,960 trials. A total of 71 participants had at least one trial dropped, but there were no more than four trials dropped for any one participant. There were two trials excluded for having RTs below 500 ms, 22 trials excluded for having RTs longer than 60 s, and 93 trials excluded for having RTs that were longer than 15 s *and* more than 3 SD higher than the participant’s mean RT.

Note that if we had not opted to retain all trials with RTs under 15 s, we would have excluded an additional 145 trials. Those trials had a mean RT of 9.6 s (range = 2.4–14.8 s), and the majority (115) were trials that required visual search because there was no bounding box or the bounding box was incorrect. In addition, the lists with the highest proportions of trials in this category were those with highest model accuracy. This pattern confirmed that using a simple + 3 SD cutoff would inappropriately bias the results in the experiments that included mock ML outputs to aid the visual search task.

### Results

**Fig. 5:**
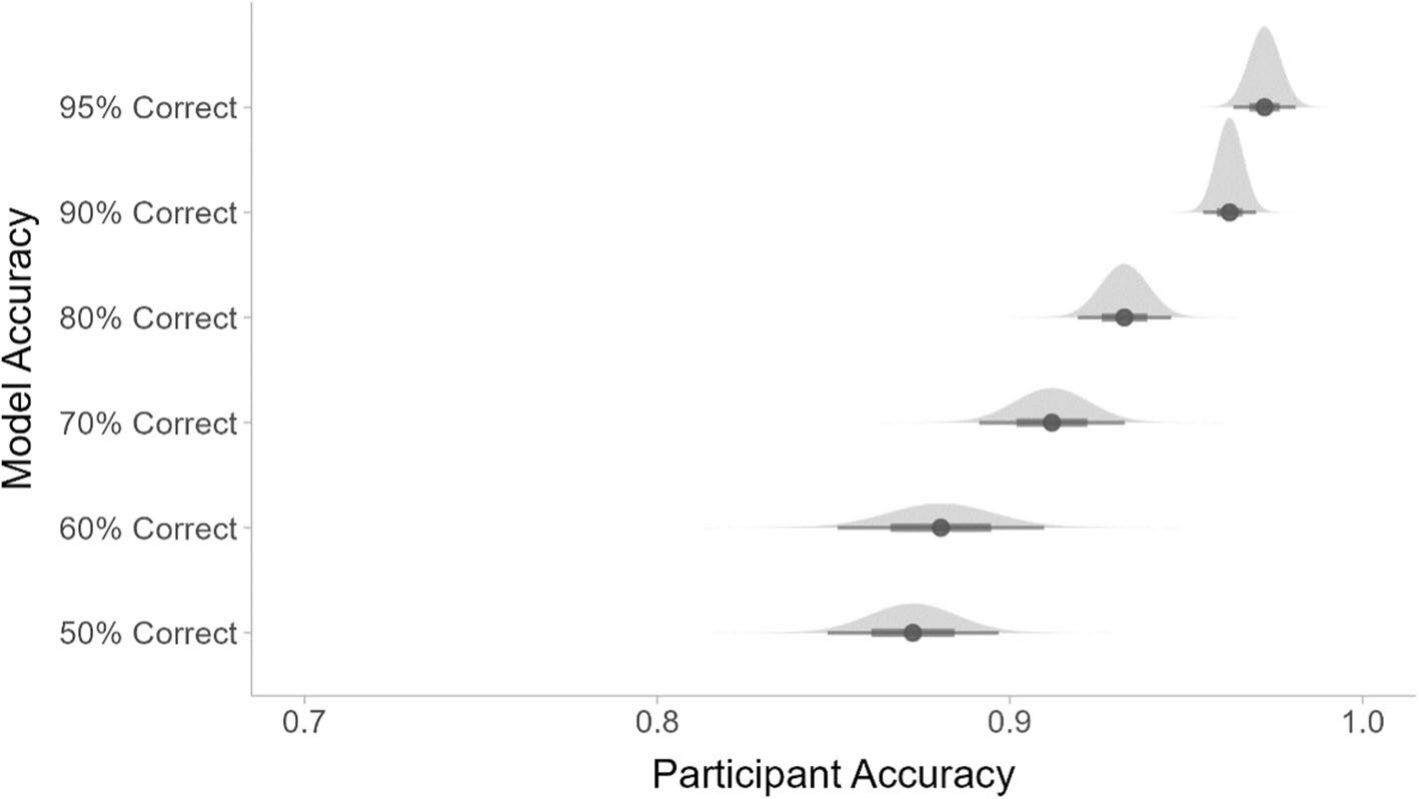
The average accuracy of the participants for each model accuracy condition

**Fig. 6:**
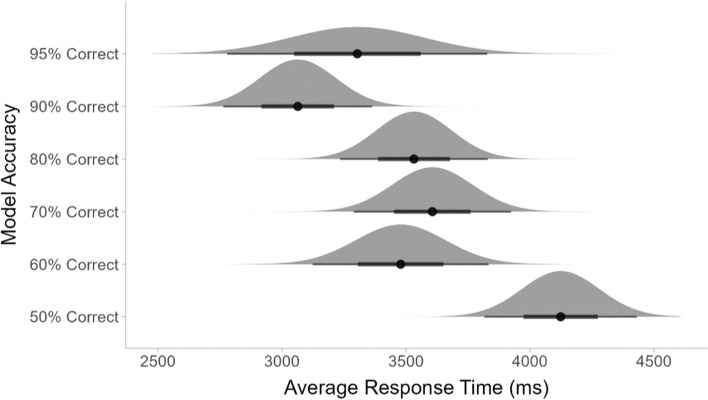
The average response times for the participants’ correct responses for each model accuracy condition

One-way ANOVAs showed that the changing model error rates had a significant impact on the participants’ overall accuracy (*F*(5,202) = 17.51, *p* < 0.001) and their average RTs for trials where they responded correctly (*F*(5,202) = 3.76, *p* < 0.01). As shown in Fig. 5, the participants’ overall accuracy increased as the accuracy of the mock ML outputs increased. Their RTs decreased when the model outputs were more accurate, as shown in Fig. 6.

**Fig. 7:**
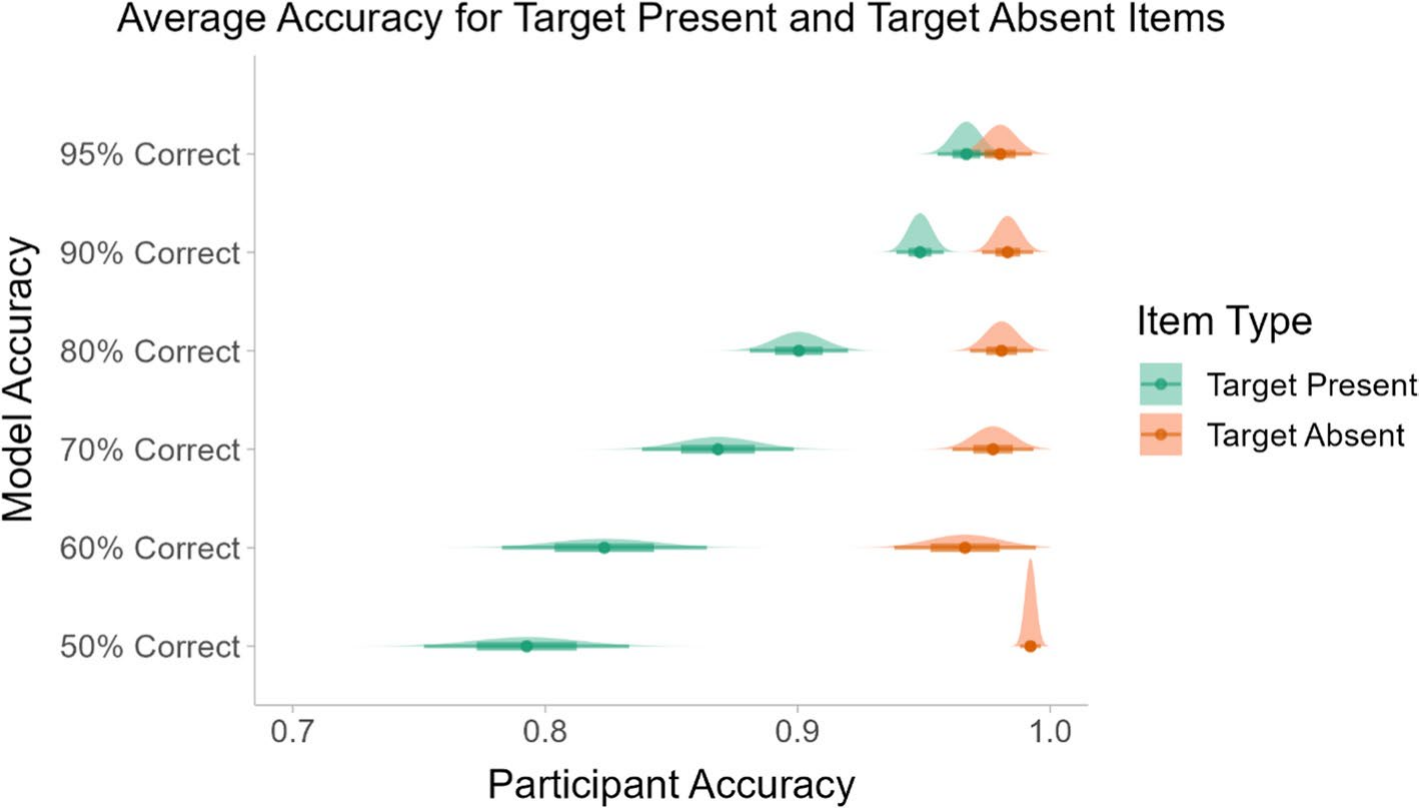
The average accuracy for target present and target absent stimuli across all model accuracy conditions

As in Experiment 1, we looked at the participants’ responses to target present and target absent trials. Their average accuracy for both types of items is shown in Fig. 7. The participants’ performance was near ceiling for the target absent items, regardless of the model accuracy condition. For the target present items, the participants’ accuracy increased consistently as the model accuracy increased. This indicates that the mock ML outputs were effective at helping participants to locate the targets in the images.

**Fig. 8:**
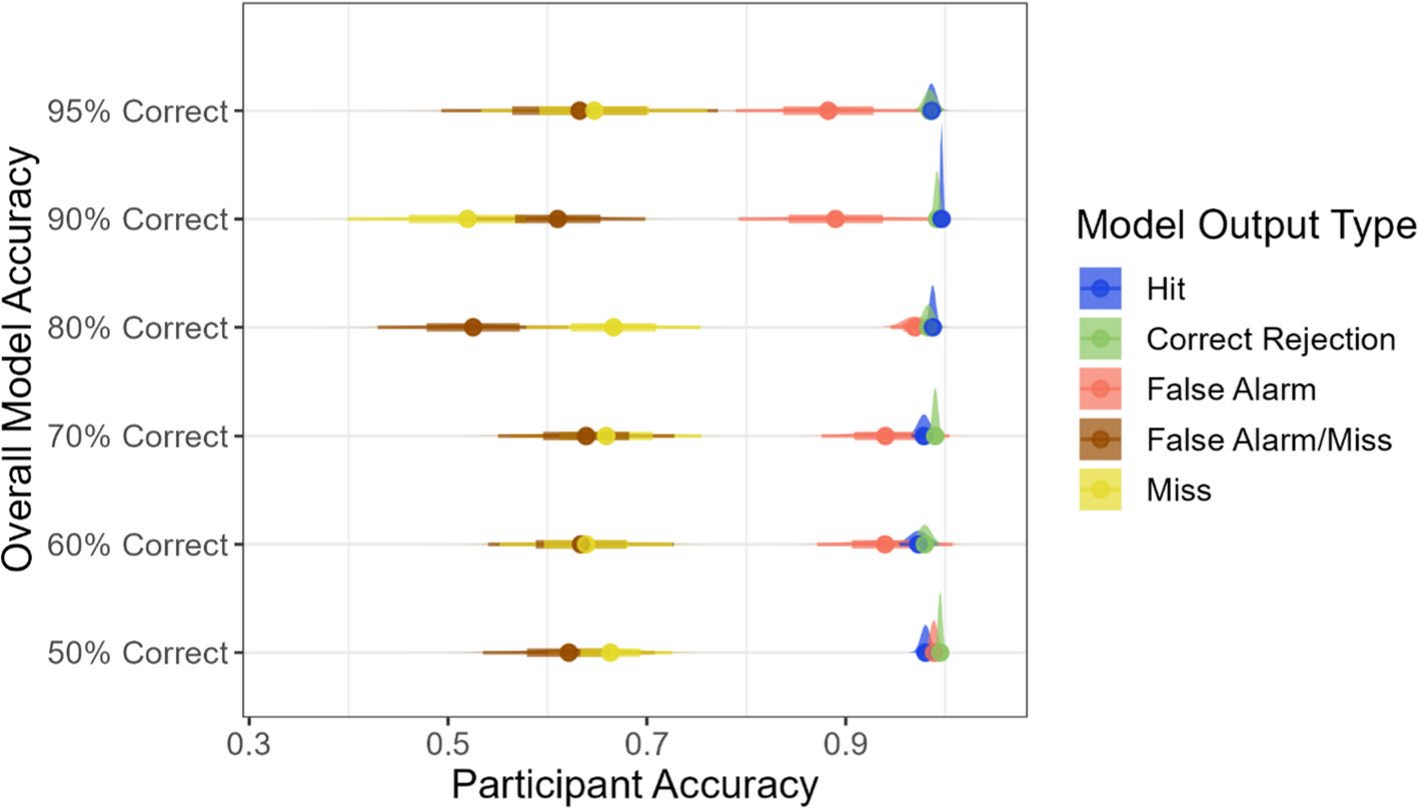
The participants’ accuracy (proportion correct) for each ML output condition across all of model accuracy conditions. Note that the number of trials in each ML error category decreases as the model accuracy increases. Moving through the model accuracy conditions from top to bottom, the number of model errors of each type (FA, miss, and FA/miss) was 2, 4, 8, 12, 16 and 20

The key question for Experiment 2 was how the changes in the accuracy of the bounding boxes would impact participants’ performance, particularly for the trials where the bounding boxes were incorrect. Those results are shown in Fig. 8. When the model outputs were correct (in the hit and correct rejection trials), the participants’ accuracy was near perfect. For these trials, the participants correctly agreed with the model outputs.

When the images contained a target, but the model output was incorrect (miss and FA/miss trials), the participants’ performance was quite poor. Their average accuracy for these trials was less than 70% correct across all of the model accuracy conditions. This indicates that the participants frequently failed to find the targets when the bounding box was absent or placed incorrectly. Notably, when the ML output missed a target, the participants in Experiment 2 were *less* likely to find that target than the participants in Experiment 1, who had no ML outputs to help them.

An interesting pattern emerged for the false alarm trials, where there was no target present in the image, but a bounding box appeared around one of the distractor items. In this case, the participants simply had to look at the letter inside the bounding box, determine whether it was a T or and L, and respond accordingly. In the conditions where the model’s overall accuracy was low, the participants generally succeeded at this task and had near-ceiling accuracy for the false alarm trials. However, as the model accuracy increased, the participants’ performance in this condition began to decrease. The participants’ accuracy was significantly lower for the FA trials in the 95% correct condition relative to the 50% correct condition (*t*(35) = 2.23, *p* < 0.05). This suggests that the participants became more complacent when the model made very few errors and they failed to adequately check the model outputs, leading them to overlook model errors that should be easy to identify.

#### Participants’ estimates of model performance

**Fig. 9:**
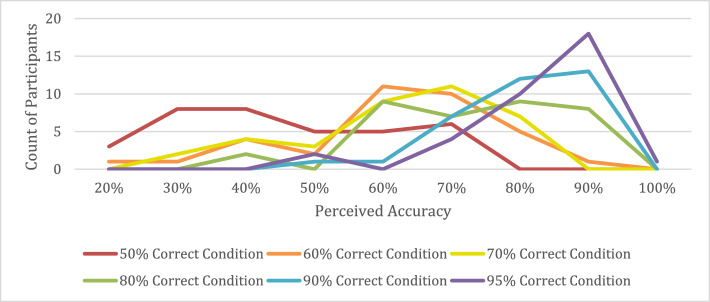
The participants’ estimates of model accuracy for each condition

At the end of the experiment, the participants were asked to estimate to overall accuracy of the model. Their responses to the perceived accuracy question are shown in Fig. 9. A total of 68 participants correctly guessed the model’s accuracy, while 42 overestimated the model accuracy and 98 underestimated the model accuracy.

Among the participants who saw the 50% correct model, a fairly large number overestimated the model’s performance. As an exploratory analysis, we compared the performance of participants who overestimated the accuracy of the bounding boxes to those whose estimates were correct or too low. We found that all three groups of participants performed equally well (all 98.8% correct) when the bounding boxes were correct (hits and correct rejections). When the bounding boxes were incorrect (FA, miss, and FA/miss trials), the participants who overestimated the accuracy of the bounding boxes had an average accuracy of 67.0% (SD = 15.9%), while participants who estimated correctly had an average accuracy of 85.0% (SD = 9.2%) and those who underestimated had an average accuracy of 78.5% (SD = 12.5%). In other words, when the mock model outputs were incorrect, the participants who overestimated the accuracy of the model performed significantly worse than those who estimated correctly (*t*(13) = 2.85, *p* < 0.01) and those who underestimated the model’s accuracy (*t*(17) = 2.05, *p* < 0.05). This effect was largely driven by the miss and FA/miss trials, since all of the participants performed near ceiling for the FA trials in the 50% correct condition. The participants correctly evaluated whether or not the letter inside of the bounding box was a T, but when they overestimated the accuracy of the bounding boxes, they failed to search the rest of the image. They were more likely to miss the cases where a T was present, but not correctly marked by the bounding box. For the other model accuracy conditions there were not enough participants who overestimated the model’s performance to conduct similar analyses.

### Discussion

The results of Experiment 2 showed that changing the accuracy of a mock ML model’s output impacted the participants’ overall accuracy and RTs as well as the types of errors that the participants made. As the accuracy of the bounding boxes increased, participants had higher performance overall, with higher average accuracy and faster RTs. This was driven by having a higher overall number of correct trials to evaluate. At the same time, as the model’s accuracy increased, participants became more likely to respond incorrectly to FA trials. This indicates that after seeing high numbers of correct hits, participants were not carefully evaluating the letter inside of the bounding boxes to ensure that it was a target. We saw the opposite pattern at the other end of the model accuracy spectrum. When the model performed poorly, participants who recognized that poor performance were more likely to identify misses and FA/misses. This indicates that they searched the images thoroughly, even when the bounding box was absent or placed around a distractor item. The participants who overestimated the accuracy of the model had much poorer performance for the miss and miss/FA items, indicating that they were relying too heavily on the bounding boxes and were not searching the images very thoroughly when the bounding box did not appear or did not contain a target.

Experiment 2 demonstrated that increasing model accuracy is beneficial to users whenever the model outputs are correct. However, it may make users less likely to notice rare model errors. This is reminiscent of the prevalence effect, where people are less likely to notice rare events or targets (cf. Wolfe et al., 2005). In the context of ML, where developers aim to maximize the performance of their model, errors can be extremely rare. However, no model is perfect. Our findings indicate that there is a real risk that users will fail to notice those rare model errors.

In Experiment 2, the three different types of model errors occurred equally often. In real-world applications of ML, different types of model errors may vary in importance and may differ in prevalence. Some algorithms make very few misses and many false alarms, while others have few false alarms and many misses. The importance of misses and false alarms can also vary by domain. Missing a target such as a weapon in a suitcase or a tumor in a mammogram can have much higher consequences than a false alarm that results in additional screening.

We sought to address questions related to differences in error type and error importance in Experiments 3–5. Experiment 3 manipulated the types of errors made by models with different overall accuracy levels. Experiments 4 and 5 manipulated the importance of different types of errors, as well as the emphasis on human performance or ML performance.

## Experiment 3: manipulating model error type

In Experiment 2, all of the lists had the same proportion of errors of each type (1/3 false alarms, 1/3 misses, and 1/3 false alarm/misses). However, changes in the overall error rate changed how participants responded to these three different error types. In Experiment 3, we manipulated the proportion of the different error types to determine how models with different types of “biases” would impact participants’ performance. For example, would participants be more likely to notice misses in a condition where misses were the predominant or only type of error? Would increasing the overall model accuracy still lead to a decrease in performance for FAs if FAs were the predominant or only type of error?

Experiment 3 had 18 experimental conditions in which the proportions of each error type were manipulated for models with three different levels of overall model accuracy. Based on the results of Experiment 2, we selected 70%, 80% and 90% for the levels of overall model accuracy. Those three conditions produced a range of performance in Experiment 2, with participants having significantly higher accuracy in the 90% condition relative to the 70% (*t*(44) = 4.50, *p* < 0.001) and 80% conditions (*t*(52) = 3.84, *p* < 0.001). We did not consider using the 95% condition because there were not enough model errors to allow for a reasonable manipulation of the proportions of errors of different types. We also eliminated the 50% and 60% correct conditions due to concern that the non-master participants would be more likely to respond randomly in the conditions where the model outputs were not particularly helpful. Furthermore, the analysis of the participants’ performance on the model error trials in Experiment 2 did not reveal any notable differences between the 50%, 60% and 70% conditions, as shown in Fig. 8.

Within each of the three levels of overall accuracy, we created lists where *all* of the errors were FAs, misses, or FA/misses. Then we created three additional lists that manipulated the proportion of misses and FAs so that they had 1/3 FAs and 2/3 misses, 1/2 FAs and 1/2 misses, or 2/3 FAs and 1/3 misses (the combined FA/miss error types were not included in these lists). In each list, the number of trials with hits and correct rejections were adjusted so that the prevalence of targets was the same for all lists, with targets always appearing in 2/3 of the trials. The Supplemental Materials show how many items of each type were included in each list.

### Results

Experiment 3 used the same data cleaning process as Experiment 2. A total of 82 participants were excluded from the analysis, either because they missed three or more of the 10 catch trials (31 participants) or because their overall accuracy was less than 60% correct (51 participants). This left 587 participants total, with 29–36 participants per condition.

Trials with RTs that were unusually short or long were rejected using the same process used in Experiment 2. A total of 538 trials from 264 of the participants were rejected. The vast majority of those participants had only one or two trials rejected, but there were three participants with more than 10 rejected trials. Those participants had 13, 18, and 33 rejected trials.

#### Overall accuracy

In Experiments 3–5 we conducted two sets of analyses: one for the participants’ overall accuracy and RTs, and another for their accuracy and RTs in response to the subset of trials where the model output was erroneous. The analyses of their overall performance generally replicated the results of Experiment 2, where increases in the overall model accuracy led to increasing participant accuracy and decreasing RTs. These analyses are presented in the Supplemental Materials and are summarized in Table 3 in the General Discussion section. In the sections below, we focus on the participants’ performance for the subset of trials in which the model produced an error.

#### Participants’ responses to trials containing ML errors in lists with a single error type

**Fig. 10:**
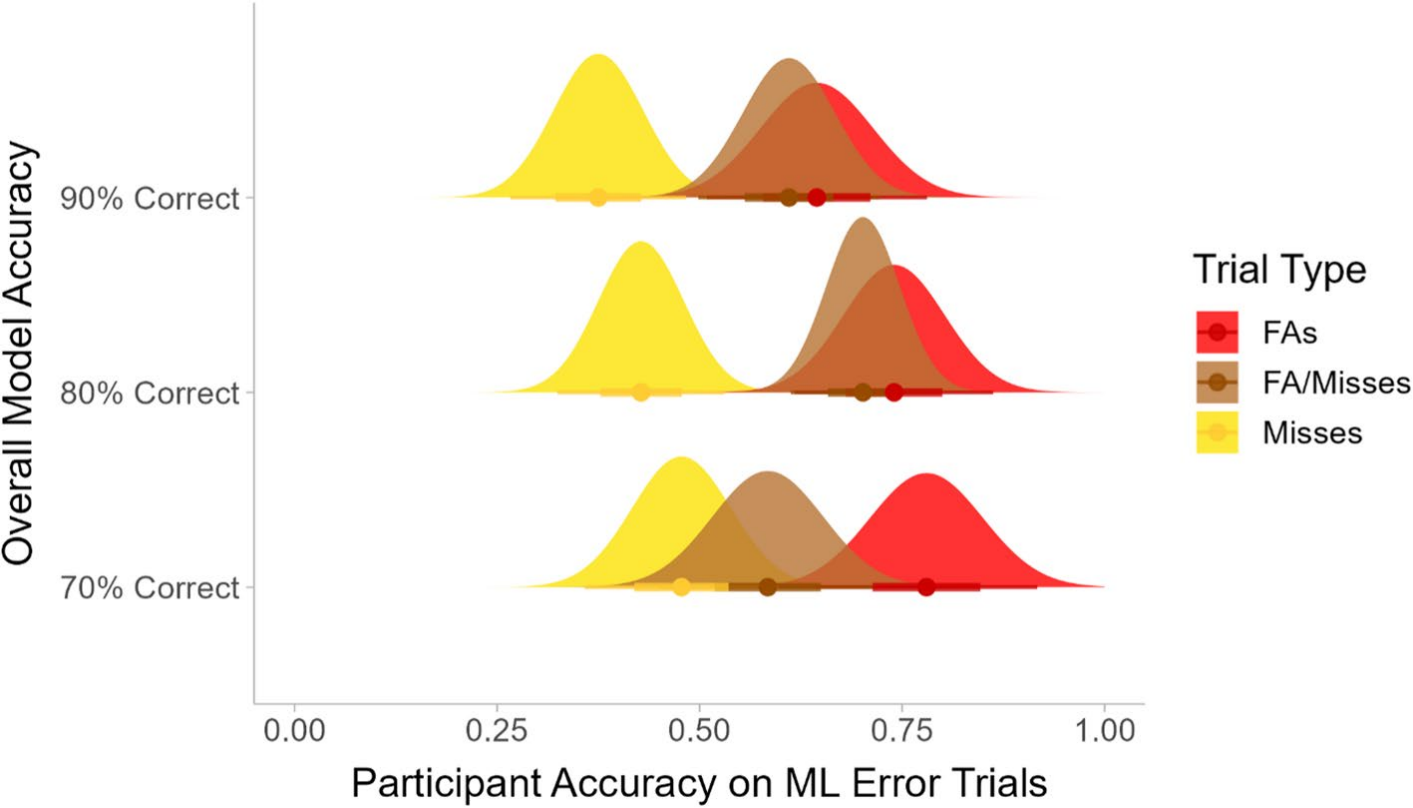
The participants’ average accuracy for trials where the model output was incorrect

In Experiment 3, our first analysis focused on the nine lists in which all of the model errors within the list were of the same type (all FAs, all misses, or all FA/misses). Figure 10 shows the participants’ average accuracy for the ML error trials in those lists. A 3 × 3 ANOVA including only the trials for which the ML output was erroneous showed that there was a significant main effect of error type (*F*(2, 293) = 18.91, *p* < 0.001), but there was not a significant main effect of overall model accuracy (*F*(2, 293) = 1.64) or a significant interaction between the two (*F*(4, 293) = 0.72). Pairwise comparisons with Bonferroni correction showed that the participants’ accuracy was significantly lower for misses than for FAs (*p* < 0.001) or for FA/misses (*p* < 0.001). There was not a significant difference in accuracy between FAs and FA/misses (*p* = 0.27).

Although there was not a significant effect of overall model accuracy, there was a downward trend for the FA and miss conditions, with participants noticing fewer of the model errors on average as the model accuracy increased. This mirrors the pattern seen in Experiment 2. In the case of the FA lists, the correct response to the model error trials was “target absent.” The bounding box marked a non-target, so participants needed to evaluate the letter inside of the box to determine whether it was a perfect “T”. This error type was the easiest for the participants to identify. The majority of the participants responded correctly on most or all of the FA trials, but a subset of the participants agreed with the model output without checking carefully. For the 70% correct list, five participants answered incorrectly on all or most (all but one or two) of the FA trials. For the 90% correct list, this number jumped to 11. This suggests that more participants became complacent when model accuracy was high and did not give close inspection to the FA trials. For the lists containing misses, the correct response to the model error trials was “target present.” The target was not marked by a bounding box, so the participants would have to search the image to discover that there was actually a target. The participants performed poorly across all of the model accuracy conditions, but they were even less likely to notice the missed targets when the model accuracy was higher overall.

**Fig. 11:**
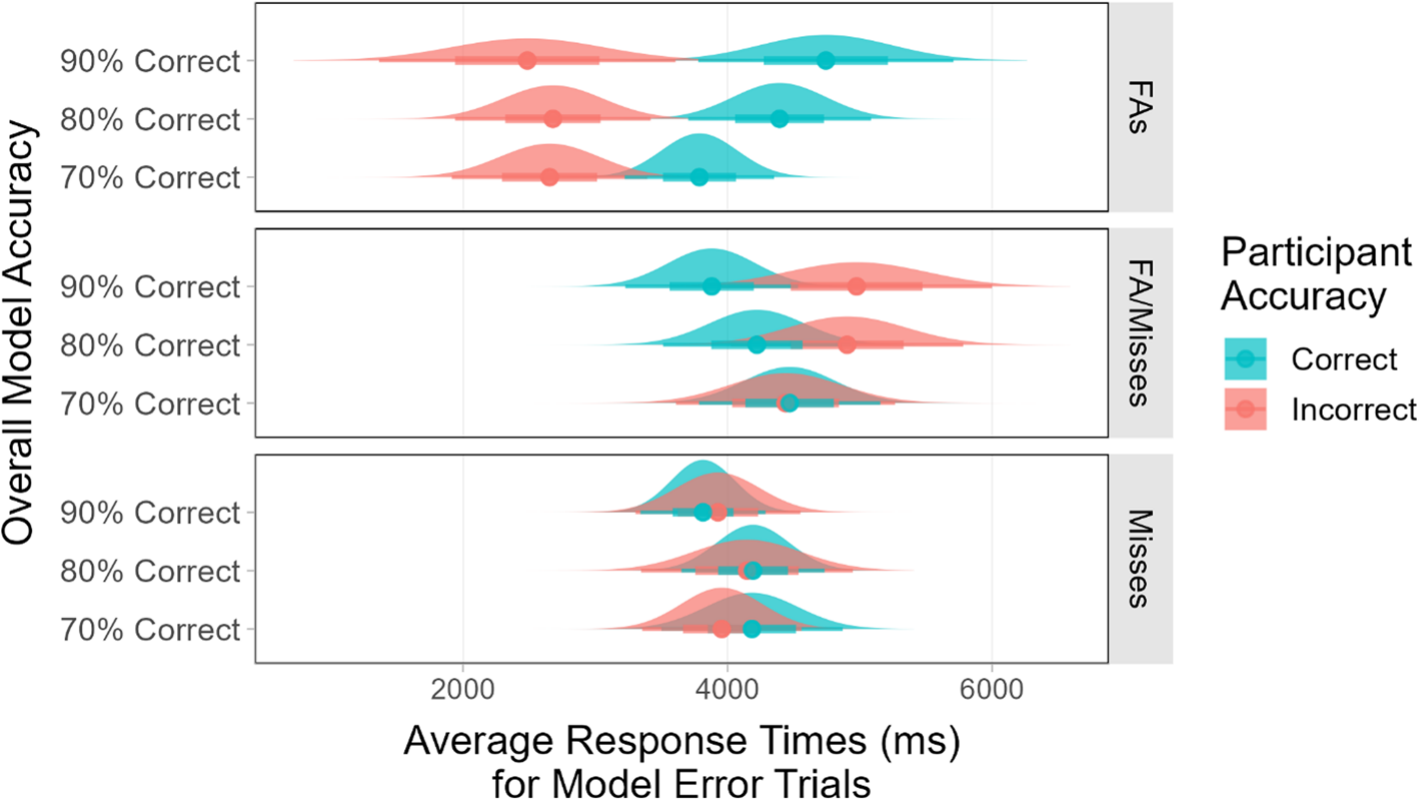
The participants’ average response times for correct and incorrect responses to model error trials

Figure 11 shows the participants' average RTs for trials where the model made an error, split based on the accuracy of the participants’ responses to those trials. When considering only the trials where the participants responded correctly, there were no significant differences in RTs for the different error type (*F*(2, 255) = 0.38) or model accuracy (*F*(2, 255) = 0.11) conditions. However, for the trials where the participants responded incorrectly, there was a significant main effect of error type (*F*(2, 243) = 17.39, *p* < 0.001). Pairwise comparisons with Bonferroni correction showed that the participants’ RTs were significantly faster when they responded incorrectly to FA trials than when they responded incorrectly to miss (*p* < 0.001) or FA/miss (*p* < 0.001) trials.

Comparisons between the correct and incorrect responses for each error type showed that for the lists that had only FAs, the participants had significantly longer RTs when they answered correctly compared to when they answered incorrectly (all *t*s > 2.38, all *p*s < 0.05). This indicates that the participants who answered incorrectly were not taking the time to verify the model’s outputs on the FA trials. Notably, the RT difference between correct and incorrect responses increased as the model accuracy increased. This suggests that some participants were more complacent when the overall accuracy of the model was high.

For the lists that had only misses, there were no significant differences in RTs when the participants answered correctly compared to when they did not (all *t*s < 1.00). The average RTs indicate that the participants were looking for missed targets, but they did not always find them.

For the lists that had only FA/misses, the average RTs were generally lower for correct than for incorrect items. When the model was 90% accurate, this difference reached statistical significance (*t*(52) = 1.76, *p* < 0.05). On the FA/miss trials, the image contained a target, but the bounding box was incorrectly placed around a distractor. There were two ways that participants could reach the correct answer for these trials. First, they could examine the letter inside of the bounding box, determine that it was not a target, and then search the rest of the image until they found the target. Second, they could note that a bounding box was present in the image and respond “target present” without careful inspection. When the FA/miss condition had an overall model accuracy of 70%, the participants had very similar average RTs for both correct and incorrect responses to the model error trials. They had relatively long RTs and relatively low accuracy. This indicates that the participants in that condition correctly recognized that the bounding box did not contain a target and that they searched for a real target in the image, but they did not always find it. As the model accuracy increased, the participants’ RTs for correct and incorrect answers began to diverge, with shorter RTs for correct responses. This suggests that some participants may have accepted the model’s output without checking it carefully, getting the response correct by accident. The more thorough participants who searched the image for a real target did not always find it and ended up responding “target absent.” However, the RT difference between correct and incorrect trials were fairly small and we cannot definitively determine whether the participants who responded correctly found the real target or simply complied with the model output.

#### Participants’ responses to trials containing ML errors in lists with different proportions of misses and false alarms

Our next analysis compared the lists that included both FAs and misses in varying proportions. Since the miss trials were consistently more difficult for participants, we expected that participants would have higher performance when there was a higher proportion of FAs among the model errors.

**Fig. 12:**
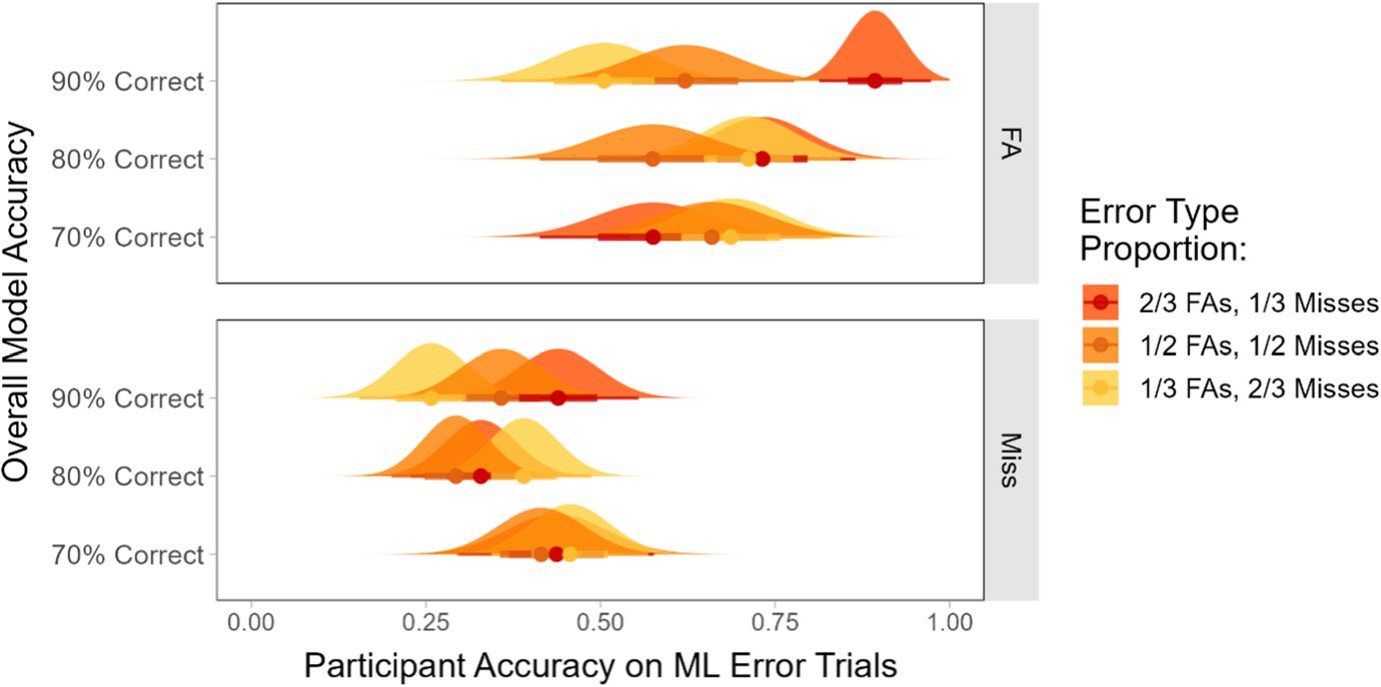
The participants’ average accuracy for the trials where the ML output was incorrect, split by the type of error the model produced on that trial

Figure 12 shows the participants’ average accuracy for the trials where the ML output was incorrect, split by the error type. A 3×3×2 mixed ANOVA (overall model accuracy by proportion of FAs by error type) showed that there was a significant main effect of error type (FA or miss; *F*(1,276) = 241.41, *p* < 0.001). There was also a significant two-way interaction between error type and overall model accuracy (*F*(2, 276) = 4.95, *p* < 0.01), a significant two-way interaction between the proportion of FAs and overall model accuracy (*F*(4, 276) = 2.94, *p* < 0.05), and a significant three-way interaction (*F*(4, 276) = 2.52, *p* < 0.05).

Simple comparisons within each level of model accuracy showed that there was a significant main effect of error type at all levels of model accuracy (all *F*s > 49.47, all *p*s < 0.001). Post-hoc tests with Bonferroni correction showed that participants had significantly higher accuracy for the FA trials than for the miss trials across all conditions (all *p*s < 0.05) except for the list where the model output was correct 70% of the time and 2/3 of the errors were FAs (*p* = 0.21).

Next, the impact of the proportion of FAs was investigated at each level of overall model accuracy. For the lists where the overall model accuracy was 90% correct, there was a significant main effect of the proportion of FAs in the model outputs (*F*(2, 93) = 7.41, *p* < 0.01) and a significant interaction between the proportion of FAs and the error type (*F*(2, 93) = 3.44, *p* < 0.05). There was a significant simple main effect for the FA trials (*F*(2, 93) = 9.10, *p* < 0.001) but not for the miss trials (*F*(2, 93) = 2.66, *p* = 0.08). In summary, when the model was correct 90% of the time, the participants were more likely to notice the ML errors and respond correctly when the proportion of FAs among the errors was higher. This trend appeared for both the miss and FA trials, but it was only statistically significant for the FA trials.

Comparing across levels of overall model accuracy showed that there was a significant effect of model accuracy for the miss trials in the condition where 1/3 of the model errors were FAs (i.e., when the majority of the model errors were misses; *F*(2, 96) = 3.62, *p* < 0.05). There was also a significant effect of model accuracy on responses to the FA trials in the 2/3 FA condition, where the majority of the model errors were FAs (*F*(2, 93) = 5.81, *p* < 0.01). Post-hoc tests with Bonferroni correction showed that when the majority of the model errors were misses, the participants were significantly worse at identifying those misses when the model was 90% correct overall than when it was 70% correct overall (*p* < 0.01). The opposite was true for the FA trials. When the majority of the model errors were FAs, the participants performed significantly *better* at identifying FA trials when the model was 90% correct overall relative to when it was 70% correct overall (*p* < 0.01).

**Fig. 13:**
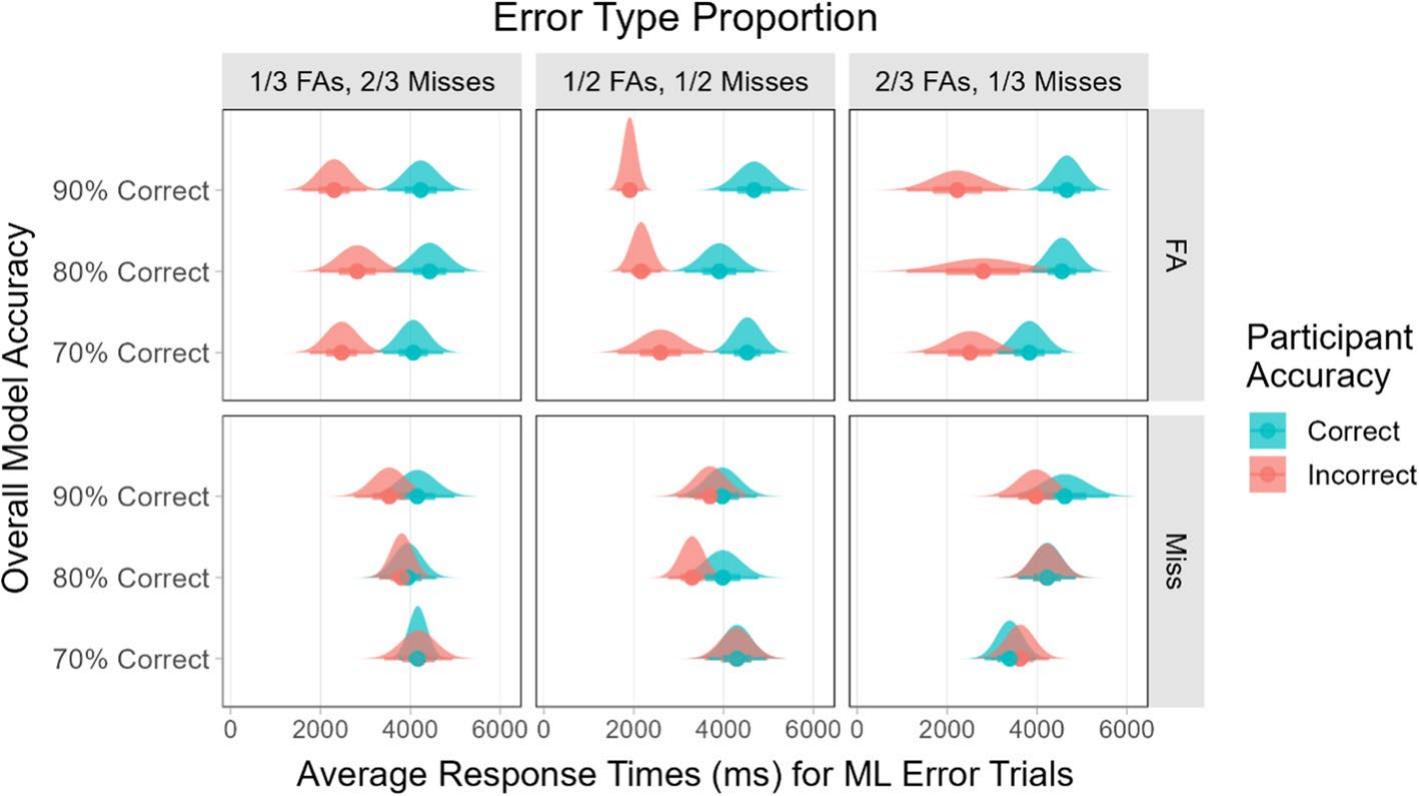
The participants’ average response times for correct and incorrect responses to model error trials under different conditions

The participants’ RTs for correct and incorrect responses for model error trials were analyzed separately. These data are shown in Fig. 13. A 3×3×2 mixed ANOVA including only the trials where participants responded correctly found a significant main effect of overall model accuracy (*F*(2, 187) = 3.40, *p* < 0.05) and a significant main effect of error type (FA or miss, *F*(1, 187) = 9.80, *p* < 0.01). Post-hoc tests showed that there was a simple main effect of overall model accuracy when 2/3 of the model errors were FAs (*F*(2, 153) = 4.11, *p* < 0.05), with the average RTs for correct responses increasing as the overall model accuracy increased. There was a significant difference in average RTs when comparing the 70% model accuracy level to the 90% model accuracy level (*p* < 0.05).

An ANOVA including only the trials where participants responded incorrectly found a significant main effect of error type (*F*(1, 151) = 23.81, *p* < 0.001) with significantly longer RTs for misses than for FAs (*p* < 0.001). This indicates that participants may have searched for an unmarked target on the miss trials, but they did not always find it.

Comparisons between the correct and incorrect responses for each error type showed that there were no significant differences in RT between correct and incorrect responses to trials where the model missed a target, regardless of overall model accuracy or the proportion of FAs among the model errors (all *t*s < 1.40, all *p*s > 0.16). For the trials where the model produced an FA, the participants always had significantly longer RTs when they answered correctly compared to when they answered incorrectly (all *t*s > 1.90, all *p*s < 0.05).

### Discussion

Just like Experiment 2, Experiment 3 showed that participants’ overall accuracy increased as the accuracy of the model outputs increased. However, our primary focus was on how participants responded to different types of errors. Note that the participants in Experiment 3 had lower overall accuracy for the model error trials than the participants in Experiment 2. Experiment 2 used participants who had a “master” qualification on Amazon Mechanical Turk, but Experiment 3 did not have that requirement due to the large number of participants needed. The participants without the master qualification had poorer overall performance on these tasks and were more likely to accept the model outputs without careful examination. However, we still observed differences in performance across the different experimental manipulations in Experiment 3.

As before, the participants struggled to identify instances where the model missed a target. They consistently had poorer performance for misses than for the other types of model errors, regardless of the model’s overall level of accuracy or of the proportion of misses and FAs among the model’s errors.

For the cases where the model produced a mix of misses and FAs, having a higher proportion of FAs was beneficial to the participants’ performance. At the highest level of model accuracy, we found that the proportion of FAs had a significant impact on performance. When there were few model errors, there was a greater risk that the participants would grow complacent and accept the model’s outputs without checking them carefully. The FAs, which were easier to notice than the misses, seemed to help participants to be more vigilant about identifying model errors. They spent more time checking the FA outputs when the proportion of FAs was higher and they consequently had higher accuracy. In addition, the RTs show that participants spent more time on the miss trials when the proportion of FAs was higher, indicating that their vigilence to model errors increased overall, not only for trials where the model placed a bounding box in the image. The opposite was true when the majority of the model errors were misses. In that case, as the model accuracy increased, the participants became less likely to find the missed targets. At the highest level of model performance, they were also less likely to notice the model’s rare FAs.

To follow-up on these results, we designed Experiments 4 and 5, where we attempted to increase the participants’ vigilence to model errors by manipulating the importance of different types of errors. In real-world ML applications, not all error types are of equal importance. In some domains, missing a target of interest, such as a tumor in a medical scan, can have catastrophic consequences. In those types of domains, algorithms might be tuned so that they have very few misses, but as a consequence they will produce more false alarms. In other domains, false alarms require a great deal of resources to address, so algorithms may be tuned to avoid false alarms at the expense of increasing the miss rate. In Experiments 4 and 5, we varied the relative importance of different types of model errors through our task instructions to participants. We expected these manipulations to change the participants’ responses to the ML error trials and their criterion (*c*; see Supplemental Materials).

Experiment 4 emphasized the importance of different types of items (target present or target absent). One condition told participants that target present images were the most important and that they should do their best to correctly categorize all of the images that contained a perfect T. We predicted that the emphasis on target present trials would lead the participants to check the contents of the bounding boxes more closely, producing better performance on the trials where the model produced a false alarm. Another condition told participants that target absent trials were most important. We predicted that these instructions would lead participants to check the images *without* bounding boxes more carefully, producing better performance on the trials where the model missed a target. The third condition did not emphasize one item type above another. The instructions simply reminded participants to correctly categorize all of the images. We expected that performance in this condition would be similar to the performance in Experiment 2, which did not give special emphasis to target present or target absent trials.

Although we expected the emphasis on target present items to improve performance on model FA trials and the emphasis on target absent items to improve performance on model miss trials, we recognized that participants might also interpret these instructions in a different way. The participants who were told that target present trials were more important might focus their attention on the images *without* a bounding box in order to search for missed targets. Similarly, participants in the condition emphasizing target absent items might pay more attention to images with bounding boxes in order to check for FAs.

Due to this possibility, we set up Experiment 5 to draw the participants’ attention to specific types of model errors. Rather then focusing on good performance on the part of the participants, the instructions in Experiment 5 shifted focus to checking the model’s performance and being on the lookout for specific types of errors. The condition that emphasized target present items warned that the model might miss targets and stated that this was the most important type of error. Participants were told to be on the lookout for model misses. The condition emphasizing target absent items stated that false alarms were the most concerning type of model error and warned participants to be on the lookout for false alarms. By shifting the focus to evaluating the model’s performance, we aimed to draw the participants’ attention to specific types of model errors and improve their performance on trials containing those types of errors.

If the participants adopted the strategies that we predicted in Experiment 4 (focusing on items with bounding boxes when target present items were deemed most important, and focusing on items without bounding boxes when target absent items were deemed most important), we would expect to see different patterns of results in Experiments 4 and 5. However, if participants adopted the alternate strategy of searching harder for missed targets when target present trials were emphasized and checking more carefully for FAs when target absent items were emphasized, then we would expect to see similar performance across Experiments 4 and 5.

Experiments 4 and 5 were designed in tandem and the data were collected at the same time with the intention of comparing across all six experimental conditions. However, for the sake of clarity, we present them as two separate experiments, with one focusing on human performance and the other focusing on model performance.

## Experiment 4: manipulating the importance of different types of items

Experiments 4 and 5 used the 70% correct, 80% correct, and 90% correct lists from Experiment 2. The only alterations were to the instructions that were given to participants prior to the task. The first four instruction screens were identical to those shown to participants in Experiments 2 and 3 (i.e., explaining that perfect Ts were the target, then showing examples of a target present trial where the bounding box was placed correctly, a target absent trial with no bounding box, and a brief explanation of the three possible ML error types). In Experiments 4 and 5, an additional screen was added to the end of the instructions, with different text for each experimental condition. The additional instruction text is shown in Table 2.

**Table 2:** The text of the last instruction screen for each condition in Experiments 4 and 5

Item type emphasis	Performance emphasis	Instruction text
Target absent images	Human performance (Experiment 4)	Remember that the machine-generated information is intended to help you complete this task, but it may not be accurate 100% of the time. Your job is to correctly indicate whether or not a perfect T is present in the image, regardless of the presence or accuracy of the bounding box indicator. **Correctly identifying all of the “Target Absent” images is the most important part of this task.** Please do your best to accurately categorize all images that do **NOT** contain a perfect T, regardless of whether the machine learning model is accurate or inaccurate
	Model performance (Experiment 5)	Remember that the machine-generated information is intended to help you complete this task, but it may not be accurate 100% of the time. Your job is to correctly indicate whether or not a perfect T is present in the image, regardless of the presence or accuracy of the bounding box indicator. **Correctly identifying all of the “Target Absent" images is the most important part of this task.** There may be cases where the machine learning model places a bounding box around something that is **NOT** a perfect T. This is the most concerning type of error for this model, since the “Target Absent” images are the most important. Please be on the lookout for this type of error. Do your best to accurately categorize all images in which there was no target, especially if the model placed a bounding box around something that is NOT a perfect T
Target present images	Human performance (Experiment 4)	Remember that the machine-generated information is intended to help you complete this task, but it may not be accurate 100% of the time. Your job is to correctly indicate whether or not a perfect T is present in the image, regardless of the presence or accuracy of the bounding box indicator. **Correctly identifying all of the “Target Present” images is the most important part of this task.** Please do your best to accurately categorize all images in which a perfect T is present, regardless of whether the machine learning model is accurate or inaccurate
	Model performance (Experiment 5)	Remember that the machine-generated information is intended to help you complete this task, but it may not be accurate 100% of the time. Your job is to correctly indicate whether or not a perfect T is present in the image, regardless of the presence or accuracy of the bounding box indicator. **Correctly identifying all of the “Target Present” images is the most important part of this task.** There may be cases where the machine learning model misses a perfect T. This is the most concerning type of error for this model, since the “Target Present” images are the most important. Please be on the lookout for this type of error. Do your best to accurately categorize all images in which a perfect T is present, especially if the model did not place a bounding box on these images or placed the bounding box in the wrong location
Both	Human performance (Experiment 4)	Remember that the machine-generated information is intended to help you complete this task, but it may not be accurate 100% of the time. Your job is to correctly indicate whether or not a perfect T is present in the image, regardless of the presence or accuracy of the bounding box indicator. **Correctly categorizing all of the images is the most important part of this task.** Please do your best to accurately categorize all images, regardless of whether the machine learning model is accurate or inaccurate
	Model performance (Experiment 5)	Remember that the machine-generated information is intended to help you complete this task, but it may not be accurate 100% of the time. Your job is to correctly indicate whether or not a perfect T is present in the image, regardless of the presence or accuracy of the bounding box indicator. **Correctly categorizing all of the images is the most important part of this task.** There may be cases where the machine learning model misses a perfect T or places a bounding box around something that is **NOT** a perfect T. These types of errors are concerning since accurate categorization is very important. Please be on the lookout for model errors. Do your best to accurately categorize all images, especially in cases where the model output is incorrect

In Experiment 4, the additional instructions emphasized that one type of item (target present or target absent) was most important, or simply reiterated that participants should do their best to correctly categorize all items. This placed the emphasis on the participant’s performance. In Experiment 5, the additional instructions called attention to specific types of model errors. This placed the emphasis on detecting errors in the model’s performance.

### Results

A total of 44 participants were excluded from the analysis, either because they missed three or more of the catch trials (13 participants) or because their overall accuracy was less than 60% correct (31 participants). This left us with 303 participants total, with 31–36 participants per condition. For the remaining participants, trials with RTs that unusually short or long were removed from the analysis, using the same procedure that was used in the prior experiments. A total of 271 trials from 148 of the participants were rejected. The vast majority of those participants had only one or two trials rejected. The highest number of rejected trials for any one participant was six.

#### Participants’ responses to trials containing ML errors

**Fig. 14:**
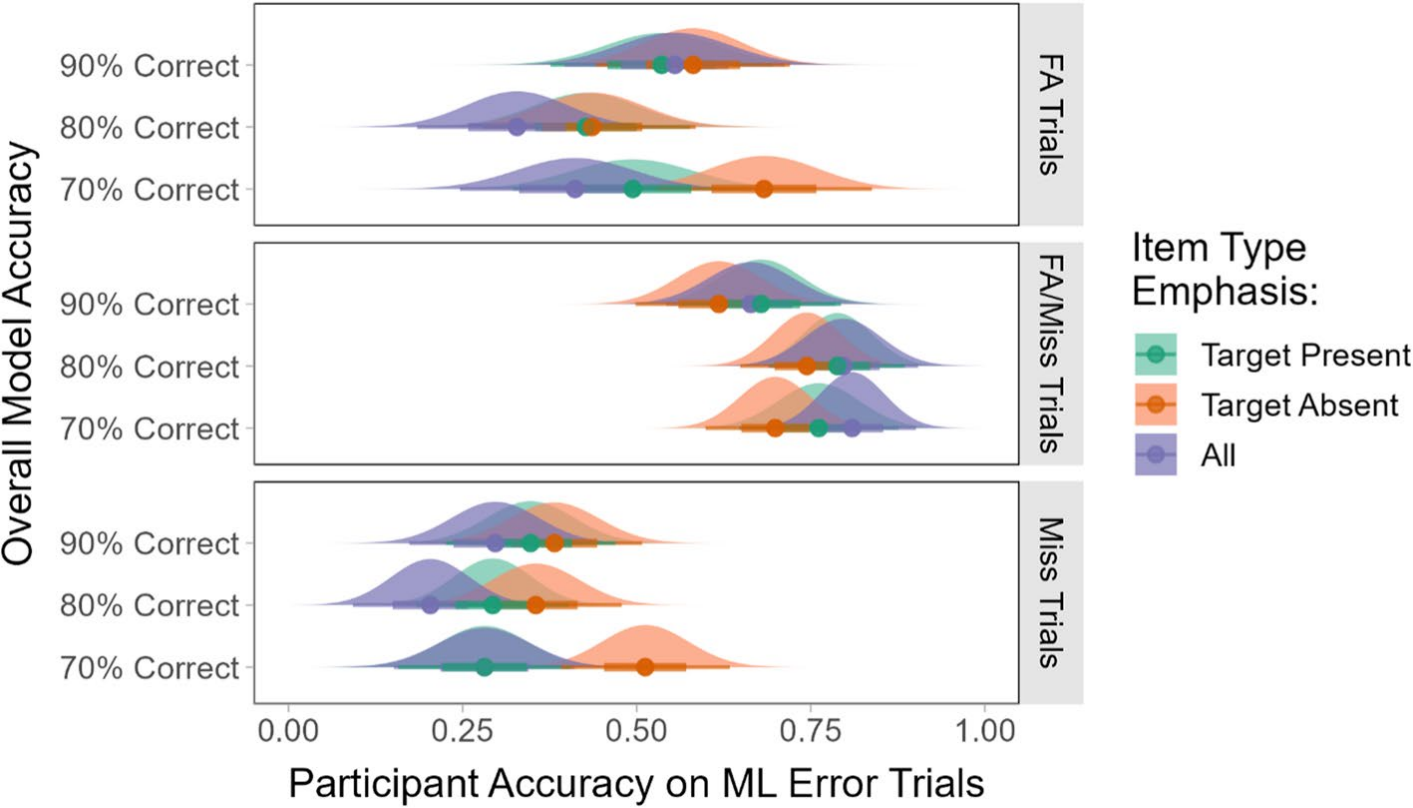
Participants’ average accuracy for trials where the ML outputs were incorrect for each error type and item type emphasis condition

As in the prior experiments, our primary interest was the participants’ responses to the trials containing ML errors. Analyses were done separately for each type of model error (FAs, misses, and FA/misses). Each analysis used a 3 × 3 between-subjects ANOVA with overall model accuracy (70%, 80% or 90% correct) and item type emphasis (greater importance given to target present items, target absent items, or neither) as the factors. We predicted that participants would perform better on the FA and FA/miss trials when they had been instructed that correct categorization of target present trials was most important. Similarly, we predicted that participants would perform better on the miss trials when they had been instructed that correct categorization of target absent trials was most important. Plots of the participants’ average accuracy in response to the model error trials are shown in Fig. 14.

The analyses found significant main effects of overall model accuracy on the participants’ ability to detect the model FAs (*F*(2, 294) = 3.66, *p* < 0.05) and FA/misses (*F*(2, 294) = 4.41, *p* < 0.05). For those types of model errors, there were no significant main effects of item type emphasis and no significant interactions (all *F*s < 2.22, all *p*s > 0.11). Pairwise comparisons with Bonferroni correction showed that the participants’ accuracy was significantly lower on the FA trials in the 80% overall model accuracy condition relative to the 90% condition (*p* < 0.05). The opposite was true for the FA/miss trials, where their accuracy was significantly lower for the 90% condition relative to the 80% condition (*p* < 0.05). There were no other significant differences (all *p*s > 0.06).

The results were different for the trials where the model missed a target. For those trials, there was a significant main effect of item type emphasis (*F*(2, 294) = 5.01, *p* < 0.01). There was not a significant main effect of overall model accuracy nor a significant interaction (all *F*s < 1.22, all *p*s > 0.30). Pairwise comparisons with Bonferroni correction showed that the participants had significantly higher accuracy for trials where the model missed a target in the condition that emphasized the importance of target absent trials relative to the condition that emphasized good overall performance (*p* < 0.01). There were no other significant differences (all *p*s > 0.11).

Similar analyses were conducted for the participants’ RTs in response to model error trials. These analyses were run separately for trials where the participants responded correctly or incorrectly. For the FA and FA/miss model errors, there were no significant main effects or interactions for either correct or incorrect responses (all *F*s < 2.26, all *p*s > 0.11). Plots of these results can be seen in the Supplemental Materials.

**Fig. 15:**
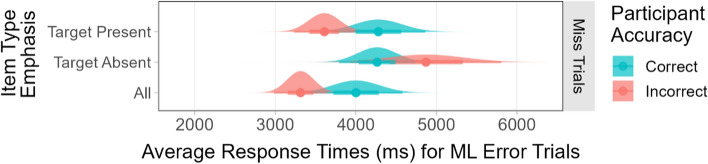
The participants’ average response times for correct and incorrect responses to ML miss trials when the instructions emphasized the importance of target present items, target absent items, or all items

Once again, the results were different for the trials where the model missed a target. When the participants responded correctly to the miss trials, their average RTs were very similar across conditions. The RT analysis for correct responses found no significant main effects and no interaction between overall model accuracy and item type emphasis (all *F*s < 2.10, all *p*s > 0.08). However, the analysis of the RTs for *incorrect* participant responses found a significant main effect of item type emphasis (*F*(2, 273) = 6.74, *p* < 0.01). When the instructions emphasized the importance of target absent trials, the participants’ RTs to incorrect trials were significantly longer than they were when the instructions emphasized target present trials (*p* < 0.05) or good performance in general (*p* < 0.01). These results are shown in Fig. 15.

### Discussion

The results of Experiment 4 suggest that instructing participants that target absent trials were most important led them to devote more time to images that did not contain a bounding box. When looking at all trials in the experiment, they spent longer on the target absent trials relative to participants who had not been instructed that one type of error was more important than another (see the Supplemental Materials for this analysis). These participants also had higher accuracy on the trials where the model missed a target, suggesting that they were searching the images that had no bounding box to confirm that no targets were present. Unlike the other conditions, the participants in this condition had longer RTs to model misses when they answered incorrectly than when they answered correctly. This indicates that incorrect responses were due to a failure to find the target via visual search rather than simply accepting the model’s output and moving on.

We did not see a corresponding change in performance when the target present items were most important. Taken together, the results suggest that participants interpreted the emphasis on target absent items as a cue to devote more attention to trials that did not contain bounding boxes. These instructions improved their performance on the miss trials but not the FA or FA/miss trials, which contained bounding boxes that were incorrect. Similarly, the participants seem to have interpreted the focus on target present items as a cue to focus on the trials that contained bounding boxes. Evaluating the contents of the bounding boxes was a quick and easy process relative to searching the images that did not have bounding boxes. Thus, the emphasis on target present items did not substantially change those participants’ overall task performance or their performance on trials where the model made an error.

## Experiment 5: emphasizing different types of model errors

Experiment 5 modified the task instructions to focus on model performance (identifying the model errors) rather than human performance (responding correctly regardless of the model output). We expected that this would produce a different pattern of performance than Experiment 4, where an emphasis on the target absent items led the participants to find more of the missed targets. In Experiment 5, the participants who were told that the target absent trials were most important were explicitly warned to be on the lookout for *false alarms* produced by the model. In other words, rather than trying to validate all of the target absent trials on their own, they were told to watch for cases where target absent trials had been categorized incorrectly by the model. Similarly, when target present items were most important, they were told to be on the lookout for cases where the model categorized the image incorrectly by missing a target. Thus, we expected that when the model’s performance was the focus, the participants would be more likely to notice FAs when target absent trials were emphasized and more likely to notice misses when target present trials were emphasized.

### Results

A total of 45 participants were excluded from the analysis, either because they missed three or more of the catch trials (11 participants) or because their overall accuracy was less than 60% correct (34 participants). This left us with 300 participants total, with 28–36 participants per condition. For the remaining participants, trials with RTs that were unusually short or long were rejected using the same procedure as in the prior experiments. A total of 310 trials from 156 of the participants were rejected. The vast majority of those participants had only one or two trials rejected. The highest number of rejected trials for any one participant was 16.

#### Participants’ responses to trials containing ML errors

**Fig. 16:**
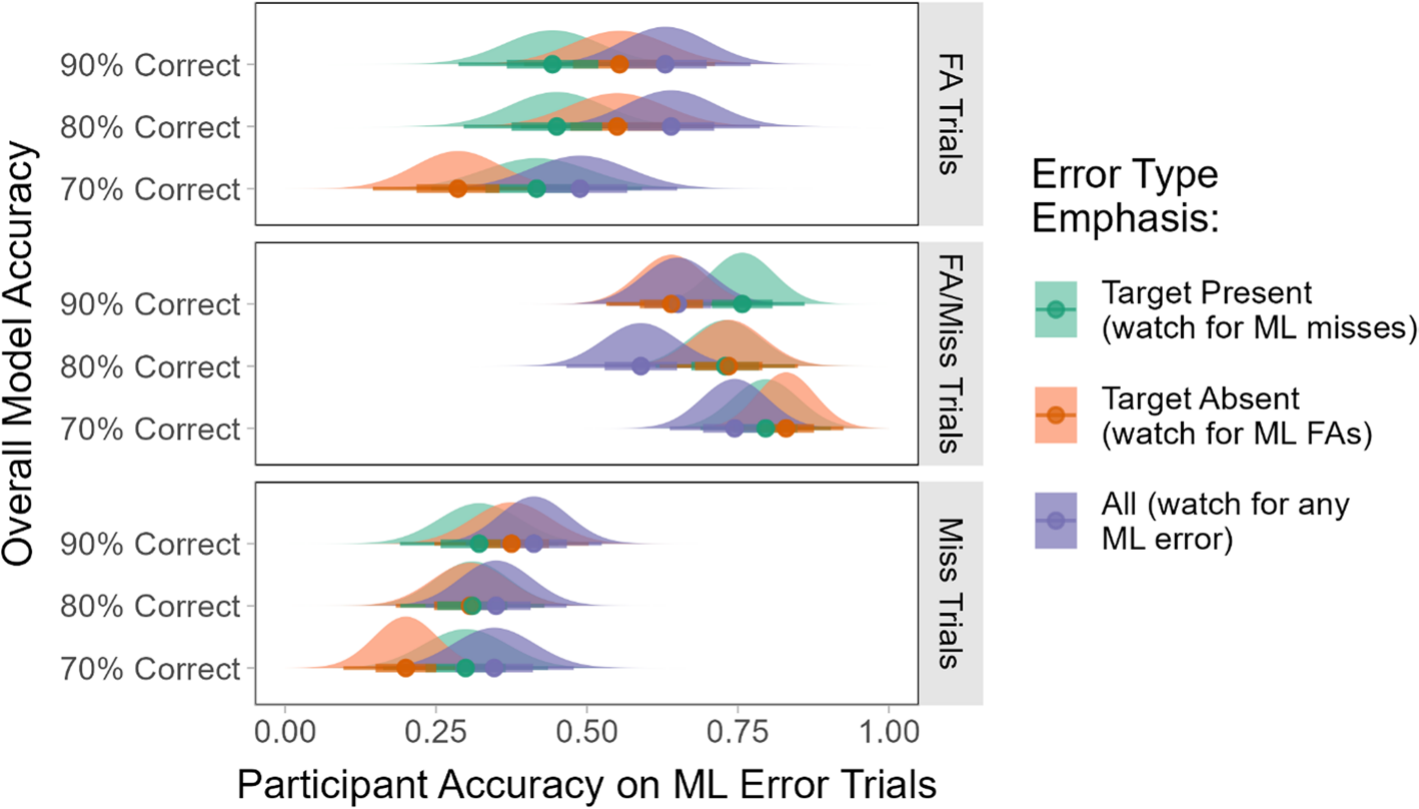
Participants’ average accuracy for trials where the ML outputs were incorrect for each error type and item type emphasis condition

We examined how the error importance manipulation influenced the participants’ performance on trials where the model made an error. This analysis was done separately for each type of model error. As in Experiment 4, each analysis was a 3 × 3 between-subjects ANOVA with overall model accuracy (70%, 80% or 90% correct overall) and error type emphasis (greater importance given to false alarms, misses, or model errors in general) as the factors. The participants’ average accuracy for the model error trials in each condition is shown in Fig. 16.

For the FA trials, there was a significant main effect of overall model accuracy (*F*(2, 291) = 3.28, *p* < 0.05) and a significant main effect of error type emphasis (*F*(2, 291) = 3.07, *p* < 0.05). There was not a significant interaction (*F*(4, 291) = 0.69). Pairwise comparisons showed that the participants’ accuracy was lower on the FA trials in the 70% overall model accuracy condition relative to the 80% (*p* < 0.05) and 90% conditions (*p* < 0.05). Participants in the condition that emphasized all model errors had significantly higher accuracy on the FA trials than participants in the condition that emphasized model misses (*p* < 0.05), but not the condition that emphasized FAs (*p* = 0.07).

For the FA/miss trials, there was a significant main effect of overall model accuracy (*F*(2, 291) = 3.34, *p* < 0.05) on the participants’ accuracy. There was not a significant main effect of error type emphasis (*F*(2,291) = 2.49, *p* = 0.08) and there was not a significant interaction (*F*(4, 291) = 0.76). Pairwise comparisons showed that the participants’ accuracy was higher in the 70% model accuracy condition than in the 80% (*p* < 0.05) and 90% (*p* < 0.05) model accuracy conditions.

For the trials where the model missed a target, the analysis found no significant effects of overall model accuracy or error emphasis on the participants’ accuracy (all *F*s < 1.44, all *p*s > 0.23).

The participants’ RTs were analyzed separately for correct and incorrect responses to each type of model error. These analyses found no significant main effects or interactions for any of the model error conditions (all *F*s < 2.03, all *p*s > 0.13). A plot of the participants’ average RTs for each condition is shown in the Supplemental Materials.

#### Comparing the conditions emphasizing model performance to the conditions emphasizing human performance

**Fig. 17:**
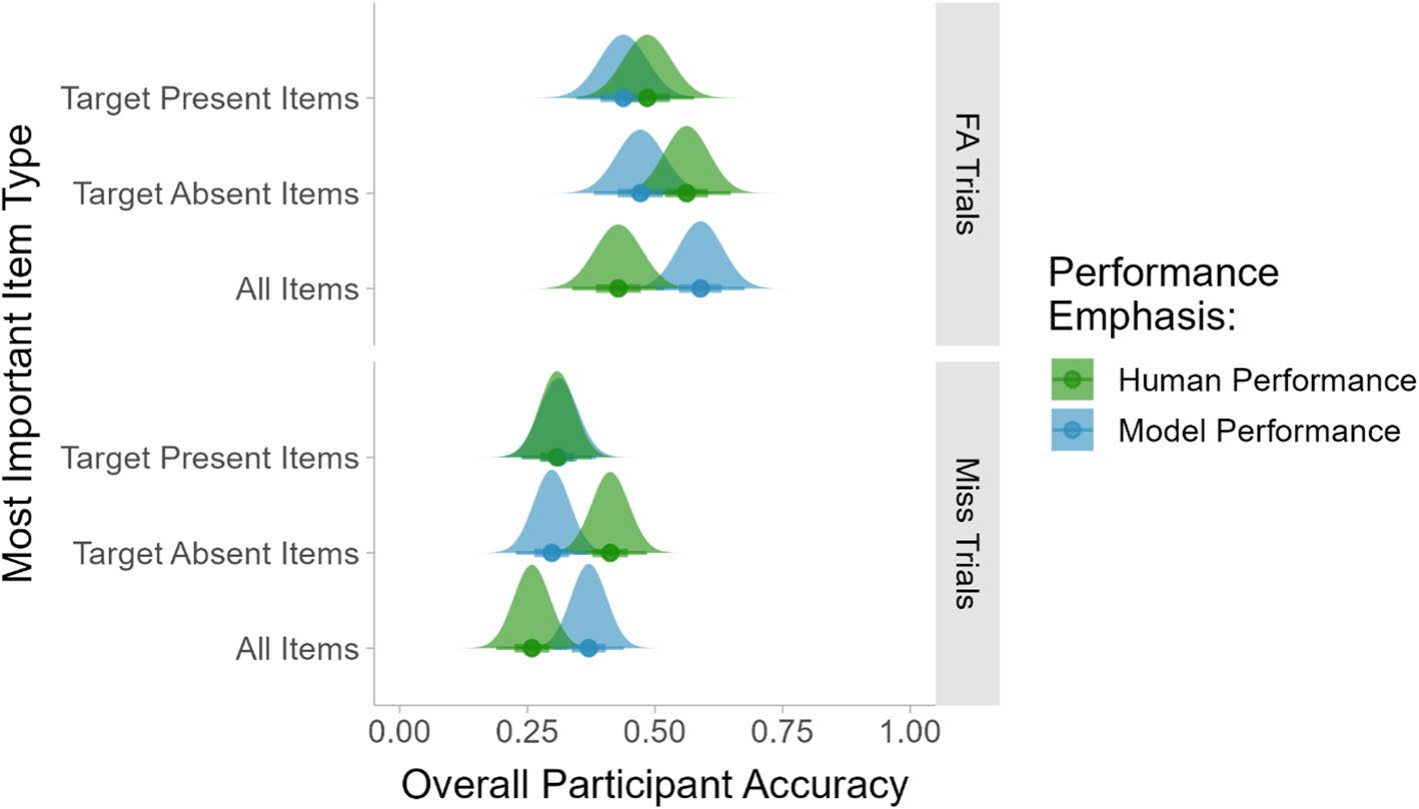
Comparisons of the participants’ accuracy to ML FAs and misses across the task conditions emphasizing human or ML performance

The data for Experiments 4 and 5 were collected simultaneously, with the intention of comparing the conditions that emphasized human performance (correct categorization of different item types) to the conditions that emphasized model performance (being on the lookout for certain types of model errors). This comparison focused on the trials where the model produced a miss or an FA. The FA/miss trials were excluded from the comparison because participants could have given the correct answer without thoroughly checking the images, which was not the case for the FA and miss trials. The analysis also collapsed across the overall model accuracy manipulation since the analyses of Experiments 4 and 5 showed no significant interactions between overall model accuracy and the task instruction manipulations. The results are shown in Fig. 17.

For the FA trials, a 3 × 2 ANOVA with item type emphasis (target present, target absent, or all items) and performance emphasis (human or model performance) as the factors found a significant interaction (*F*(2, 597) = 4.31, *p* < 0.05). Post-hoc tests with Bonferroni correction showed that when all types of items were deemed equally important, the participants performed better when model performance was emphasized than when human performance was emphasized (*p* < 0.05). When one type of item was deemed more important than the other, there were no significant differences between the human performance and model performance conditions (all *p*s > 0.16). In the conditions where all item types were given equal importance, the version of the instructions that emphasized human performance simply said, “Please do your best to accurately categorize all images, regardless of whether the machine learning model is accurate or inaccurate.” In contrast, the version that emphasized model performance said “Please be on the lookout for model errors. Do your best to accurately categorize all images, especially in cases where the model output is incorrect.” The emphasis on model errors was sufficient to improve the participants’ performance on FA trials.

For the trials where the model missed a target, a 3 × 2 ANOVA with item type emphasis and performance emphasis as the factors found a significant interaction (*F*(2, 597) = 4.98, *p* < 0.01). Post-hoc tests with Bonferroni correction showed that when all item types were emphasized equally, the participants had significantly higher performance in the model performance condition than in the human performance condition (*p* < 0.05). This mirrors the pattern observed for the FAs, indicating that a warning about model errors made the participants more likely to notice the model errors. When more importance was given to the target absent items, the participants performed better in response to model misses when human performance was emphasized (*p* < 0.05). In this case, the participants in the human performance condition were told “Please do your best to accurately categorize all images that do NOT contain a perfect T, regardless of whether the machine learning model is accurate or inaccurate.” The miss trials did not have a bounding box and would have first appeared to be target absent trials. If participants checked them carefully, they would have found the missed targets. The model performance condition emphasized the fact that the model might place a bounding box around a letter than was not a perfect T. The participants were less likely to thoroughly search the images where the model missed a target in that case.

A similar analysis comparing the participants’ RTs for correct responses across the conditions emphasizing human performance and ML performance did not find any significant differences for either error type (all *F*s < 1.00).

### Discussion

In Experiment 5, warning participants to be on the lookout for ML errors in general improved their ability to detect model FAs, even more so than warning them to be on the lookout for model FAs specifically. However, warning them about ML errors did not improve their performance on the trials where the model missed a target.

Comparisons between Experiments 4 and 5 showed that the participants had the highest performance on the miss trials when the instructions emphasized good human performance, telling them to accurately categorize all target absent items regardless of the model’s output. In that case, the participants focused on the trials that did not have bounding boxes and searched them thoroughly to ensure that there was no target. In the process, they found many of the missed targets. Telling the participants that target present trials were most important did not lead to similar improvements in performance for the FA trials, which were comparatively easy to identify to begin with.

## Summary of findings

Table 3 summarizes the participants’ overall accuracy for each of the experiments and experimental conditions used in this study. Note that all of the conditions with ML outputs led to better performance than the unaided task in Experiment 1. This is consistent with prior research indicating that computer aided detection can improve human detection performance even when the model is not always correct (Drew et al., 2012). However, it was somewhat surprising that the conditions where the model accuracy was as low as 50% or 60% correct were still beneficial to the participants. It is also notable that all four of the experiments using mock ML outputs found a significant main effect of overall model accuracy on the participants’ performance. These experiments consistently demonstrated that the more accurate models provided a larger benefit to participants.

**Table 3:** The participants’ average overall accuracy (and standard deviation) for all experiments and conditions

		No ML outputs	Mock ML outputs with an overall accuracy of:						Notes
			50%	60%	70%	80%	90%	95%	
Expt 1	Baseline	80% (10%)							
Expt 2	1/3 of errors are FAs, 1/3 are misses, 1/3 are FA/misses		87% (7%)	88% (9%)	91% (6%)	93% (4%)	96% (2%)	97% (3%)	Significant effect of overall model accuracy
Expt 3	All errors are FAs				91% (12%)	92% (11%)	93% (10%)		Significant main effect of overall model accuracy Significant main effect of error type
	All errors are misses				83% (11%)	86% (9%)	92% (4%)		
	All errors are FA/misses				87% (12%)	93% (7%)	94% (4%)		
	2/3 of errors are FAs, 1/3 are misses				82% (14%)	90% (9%)	95% (6%)		Significant main effect of overall model accuracy
	½ of errors are FAs, ½ are misses				85% (11%)	86% (9%)	92% (8%)		
	1/3 of errors are FAs, 2/3 are misses				85% (11%)	88% (7%)	92% (4%)		
Expt 4	All items important				83% (13%)	87% (7%)	92% (12%)		Significant main effect of overall model accuracy
	Target Present items most important				84% (12%)	87% (10%)	92% (11%)		
	Target Absent items most important				86% (13%)	86% (12%)	92% (8%)		
Expt 5	All model errors are important				84% (11%)	88% (10%)	91% (13%)		Significant main effect of overall model accuracy Significant main effect of item type (target absent/target present)
	Model FAs most important				80% (11%)	89% (9%)	93% (8%)		
	Model misses most important				83% (13%)	88% (10%)	94% (5%)		

Table 4 summarizes the participants’ accuracy for trials on which the model produced a miss or FA. The combined FA/miss errors are excluded from this table because they were not used in all of the experiments. The table also shows how many trials of each type were used in each condition. The Mechanical Turk master participants in Experiment 2 were substantially better at responding correctly to the model error trials than the non-master participants who were used in the other experiments. However, all of the participants in all of the experiments consistently struggled to identify the model misses. This was true even when the model only produced misses and when participants were specifically warned to be on the lookout for missed targets.

**Table 4:** This table shows the number of FAs and misses produced by the mock ML in each condition, as well as the average accuracy (and standard deviation) of the participants’ responses to those trials

			Mock ML outputs with an overall accuracy of:																					
			70%								80%								90%					
			FAs		#		Misses		#		FAs		#		Misses		#		FAs		#		Misses	#
Expt 2	1/3 of errors are FAs, 1/3 are misses, 1/3 are FA/misses		94% (20%)		12		66% (29%)		12		97% (7%)		8		67% (26%)		8		89% (29%)		4		52% (36%)	4
Expt 3	All errors are FAs		78% (38%)		36				0		74% (37%)		24				0		64% (42%)		12			0
	All errors are misses				0		48% (34%)		36				0		43% (31%)		24				0		37% (32%)	12
	2/3 of errors are FAs, 1/3 are misses		58% (45%)		24		44% (38%)		12		73% (40%)		16		33% (30%)		8		89% (23%)		8		44% (34%)	4
	½ of errors are FAs, ½ are misses		66% (44%)		18		41% (33%)		18		58% (46%)		12		30% (26%)		12		62% (44%)		6		36% (32%)	6
	1/3 of errors are FAs, 2/3 are misses		69% (43%)		12		46% (33%)		24		71% (38%)		8		39% (28%)		16		50% (43%)		4		26% (30%)	8
Expt 4	All items important		41% (48%)		12		28% (37%)		12		33% (43%)		8		20% (33%)		8		55% (46%)		4		30% (36%)	4
	Target Present items most important		49% (49%)		12		28% (35%)		12		43% (46%)		8		29% (34%)		8		54% (48%)		4		35% (37%)	4
	Target Absent items most important		69% (44%)		12		51% (34%)		12		44% (45%)		8		36% (37%)		8		58% (43%)		4		38% (38%)	4
Expt 5	All model errors are important		49% (46%)		12		35% (38%)		12		64% (44%)		8		35% (35%)		8		63% (43%)		4		41% (34%)	4
	Model FAs most important		29% (39%)		12		19% (29%)		12		55% (48%)		8		31% (37%)		8		56% (47%)		4		38% (38%)	4
	Model misses most important		41% (47%)		12		30% (37%)		12		45% (46%)		8		31% (36%)		8		44% (47%)		4		32% (40%)	4

## General discussion

The goal of this study was to investigate the impact of machine learning errors on human performance. Although there has been a great deal of prior research on the impact of errors from automated systems on human performance (cf. Dixon et al., 2007; Kunar, 2022), these topics have received relatively little systematic attention in the context of research on machine learning algorithms. We used the classic T and L visual search task as a proxy for real-world tasks in which ML algorithms could be used to provide decision support to human users. The T and L task is difficult enough to prevent perfect performance on the part of the participants, but it is also possible for participants to verify the accuracy of the ML outputs if they check carefully. The experiments used mock ML outputs, allowing for careful control of the ML output accuracy and placement within the images.

Experiment 1, which had no ML aid, served as a baseline for comparison against the subsequent experiments. In general, we found that even ML “models” with poor overall accuracy could improve participants’ performance relative to the unaided baseline condition. The targets were not easy to find, so the addition of bounding boxes substantially improved the participants’ performance on target present trials, even when the bounding boxes were only correct half of the time. The participants made good use of the outputs when the bounding boxes were correct.

As the overall accuracy of the ML outputs increased, the participants’ overall accuracy increased as well. Their average RTs decreased since it was faster and easier to evaluate a correct bounding box than to search for unmarked targets. However, Experiment 2 showed that as the model accuracy increased, the participants became more likely to overlook the rare instances where the model made an error. This effect was especially notable when the model produced a false alarm by placing a bounding box on an image that did not contain a target. The model FAs should have been easy to evaluate—participants simply needed to check the letter inside the bounding box to see if it was a T. Yet as the model accuracy increased, the participants were more likely to accept its outputs without checking them carefully. This led to higher error rates on the part of the participants in response to the model FA trials. There was little or no decrement in performance in response to the model’s misses, which were very difficult for participants to detect under all circumstances.

Our findings in Experiment 2 are consistent with the prior literature on the difficulty of finding rare targets (Horowitz, 2017; Hout et al., 2015; Peltier & Becker, 2016; Rich et al., 2008; Wolfe et al., 2005). As the model accuracy increased, the errors were increasingly few and far between, and the participants were less likely to notice them. In this case, this effect was also driven by increasing compliance with the ML outputs. These results are also consistent with prior studies showing that participants come to rely on automated systems such as CAD, making them more likely to miss targets that the CAD system has missed (Drew et al., 2012; Kunar, 2022; Kunar et al., 2017a, 2017b). Prior studies have also shown that participants are more likely to comply with ML outputs when they have been told that the model has high accuracy or when the outputs indicate that the model has high confidence (Rechkemmer & Yin, 2022; Suresh, Lao & Liccardi, 2020). In this experiment, the participants’ average RTs suggest that they complied more with the ML outputs when the model had higher accuracy overall. In this situation, they rapidly accepted the outputs and moved on to the next trial without examining the images carefully. This pattern is also consistent with prior work using a different type of visual inspection task (Matzen et al., 2019, 2021b). In that case, when the formatting of two lists supported rapid comparisons between them, the participants were more likely to overlook subtle errors. In the present study, the highly accurate ML outputs supported rapid task completion, but this came at the cost of overlooking easy-to-detect false alarm trials.

Note that the relationship between overall model accuracy and participants’ performance on the ML error trials was less clear in our subsequent experiments. This could be due to the fact that the participants in Experiment 2 had a masters qualification from Amazon Mechanical Turk, reflecting good performance across a wide variety of tasks, while the participants in Experiments 3–5 were not required to have this qualification. Although some prior studies have found comparable performance between masters workers and non-masters workers on MTurk (Loepp & Kelly, 2020; Rouse, 2019), we found that the non-masters workers generally had lower accuracy in response to the ML error trials. Since their accuracy was lower overall, there was not always a decrement in performance for the ML error trials as the overall model accuracy increased.

Experiment 3 found that participants performed better when the proportion of FAs was higher. Higher performance for the FAs themselves was not surprising because this was the easiest type of error to identify. However, there was also a trend toward better performance on the miss trials when there was a higher proportion of FAs among the model errors. This suggests that the higher proportion of FAs made participants more likely to notice model errors in general, especially in the 90% model accuracy condition, where any type of error was rare. Conversely, when most of the errors were misses, the participants did poorly on the model error trials overall and tended to have lower performance on the FA trials in addition to the miss trials.

The manipulations of the relative importance of different types of items in Experiment 4 was intended to reflect real-world settings, where some types of items are often more consequential than others. When we told participants that the target absent images were most important, we expected to see one of two outcomes. First, participants might focus on checking the contents of the bounding boxes to ensure that they were correct and that target absent trials were not being miscategorized by the model. This approach should lead to better performance on the FA and FA/miss trials. Second, the participants might focus on the trials that did *not* contain a bounding box to verify that they were indeed target absent trials. This approach should lead to better performance on the miss trials. Although individual participants may have taken the first approach, the average performance of the participants suggests that they generally took the second approach. Emphasizing the importance of target absent trials led to improved performance on the trials where the model missed a target. Participants who were told that target present trials were most important had lower accuracy for the model misses. The results suggest that these participants tended to focus on the trials containing bounding boxes. However, since the FAs were easy to identify in general, emphasizing the target present trials did not improve the participants’ performance for either FAs or FA/misses.

In Experiment 5, we modified the instructions to emphasize specific types of model errors. In the condition where the target absent trials were most important, the participants were told to watch out for FAs produced by the model. This was an attempt to move participants toward the first strategy outlined above: checking the contents of the bounding boxes to ensure that the model had not miscategorized a target absent item. However, these instructions had little impact on the participants’ performance for the model FAs. Exhorting the participants to look out for model errors in general led to improved performance for both FAs and misses, but warning them about one type of error or the other did not improve their performance for that error type. This is consistent with the findings of Kunar and Watson (2023), who manipulated whether participants were warned about the drawbacks of relying on a CAD system. They found that warning participants that use of the CAD outputs might lead them to miss a cancer had little effect on the participants’ performance. However, a more severe warning, telling the participants not to use the CAD cues at all, led to improved human performance on the trials where the outputs were incorrect.

### Limitations

One of the key limitations of this study is the small number of trials in some of the model error conditions, as shown in Table 4. Our experimental design manipulated the model accuracy and the proportion of different types of model errors while keeping the total number of trials constant. This had the advantage of keeping the length of the task constant for all participants, but it had the disadvantage of small trial counts in some conditions. In future work, it would be beneficial to construct a longer task that would allow for higher trial counts in the rare error conditions and greater statistical power.

Our experiments also raise questions about the local effects of model errors. When participants noticed a model error, they may have been more vigilant on subsequent trials. In contrast, when the model outputs were highly accurate and the gaps between model errors were long, the participants may have become more complacent over time. Our experiments presented the trials in a different random order for each participant, so it is difficult to analyze the local effects of trial type on the participants’ responses. Future work could manipulate both the number of model errors and the number of intervening trials between them to test for local effects of model errors on the participants’ vigilance or complacency.

Finally, a major limitation of this study is that the participants had little motivation to perform well on the task. The behavior of these online participants is likely to be different from that of motivated users who are performing a high-consequence task. In future research, this limitation could be addressed through the use of bonus payments tied to good performance. For example, would participants do better at identifying model misses if they had a monetary incentive for each model error they identified? Another way to address this limitation would be through the use of more realistic tasks with participants who are experienced with the task and motivated to perform well (e.g., Drew et al., 2012). This set of experiments was designed to gain a better understanding of the impact of model errors on user performance across a broad range of conditions. Despite the low stakes of the experiments, we identified several consistent findings that have implications for human-ML interactions and that lay the groundwork for future research with real-world ML tools and tasks.

### Implications for future research and development

Our findings have several implications for the design of decision support systems and also for future research on human-ML systems. One take-away from this study is that models with relatively low performance may be acceptable in some circumstances. For tasks that are difficult for humans, such as finding a difficult-to-see target, a model that only finds half of the targets can still boost the overall performance of the human-ML team. So long as the human user has appropriate expectations about the model’s performance, an inaccurate model can be useful. The user’s expectations and awareness of the ML’s errors are an important piece of this puzzle. For example, when participants in Experiment 2 used a model that was correct 50% of the time, the participants who did a better job of identifying the model errors also formed an accurate impression of the model’s accuracy. Other participants noticed fewer errors and therefore overestimated the model’s performance.

A second key finding is that rare ML errors are more difficult for people to detect. This finding mirrors the prior research on the difficulties inherent in finding rare targets. In the case of ML-based decision support tools, where it may be very important for users to notice any ML errors, the system must provide users with some form of support to help them to notice these rare occurrences. Our experiments consistently found that people struggled to notice the errors produced by high-performing models, particularly in the case of missed targets, which were the most difficult type of ML error to identify. Warning the participants to watch out for these rare and difficult-to-detect errors did nothing to help their performance (although there was some benefit to warning participants to watch out for ML errors in general). Future research should investigate whether different types of warnings or other forms of user support would provide a bigger benefit to users. This framing could be useful for research on ML transparency and explainability. Although there is a great deal of research on those topics, relatively little of it focuses on the cognitive needs of an end user. The studies that have tested the impact of transparency and explainability on users have found that the hypothesized benefits do not always materialize (cf. Bruzzese et al., 2020; Coppers et al., 2018; Kizilcec, 2016). Attempts to give users greater insight into how a model works may be ignored (Nyre-Yu et al., 2021) or they may lead users to trust an ML model inappropriately (Eiband, Buschek, Kremer & Hussman, 2019), sometimes to the point of trusting the model’s outputs above their own judgements (Suresh et al., 2020). Thus, although numerous methods for increasing ML transparency and explainability have been proposed, it is unclear how well those methods would help an end user to accurately identify errors produced by a decision support tool. This is an area in need of a great deal of additional research.

Another avenue for future research is to test human-ML performance and users’ ability to detect ML errors in situations where the stakes are higher. In the present studies, the task was quite abstract, and the participants had little motivation to perform at their best. We might see different patterns if we raised the stakes. A simple way to do this would be to provide monetary incentives for correct decisions. Experiments using real-world tasks or domain experts might also find different patterns of results. Raising the stakes for these types of experiments would also be useful for extending the current research on trust in AI. Although there are several definitions of trust in the context of human use of AI/ML tools, most definitions consider risk to be a key component of trust (Jacovi et al., 2021; Lee & See, 2004; Mayer et al., 1995). If there is no risk involved in a decision, there is no need for trust. Our experiments did not test the users’ explicit trust in the various instantiations of our mock model, but the results suggest that the participants were more likely to comply with the outputs of the higher performing versions of the model. However, without any risk associated with the decisions, these patterns cannot be used to infer higher levels of trust on the part of the users.

Another important finding from this study is that ML misses were consistently (and substantially) more difficult for users to detect relative to FAs. This difference is partly due to the nature of the stimuli, but it is likely to be true of other decision support contexts, particularly those that involve detection or identification of some target of interest. The fact that humans struggle to detect model misses has implications for system design. For example, target detection or identification systems could provide better support to users by marking possible targets even if the model’s confidence is relatively low. This could help to play to the human user’s strengths and lead to improvements in overall human-system performance. However, false alarms can also be detrimental to human performance (Dixon et al., 2007), so the appropriate balance between avoiding misses and minimizing false alarms is likely to differ for different types of tasks and systems.

In conclusion, research on ML performance, explainability, transparency, and trust must keep the cognitive needs of the user at the forefront. These studies used tightly controlled experimental stimuli and mock ML outputs to identify themes in human responses to ML errors. Those themes can help to guide future research and development for decision support tools that incorporate ML.

### Supplementary Information

**Supplementary file 1. **

## Data Availability

The materials and data from all of the experiments presented in this paper are available upon request.
