# Supplementary material for: Effects of machine learning errors on human decision-making: manipulations of model accuracy, error types, and error importance
Source: Cogn Res Princ Implic. 2024 Aug 26;9:56. doi: 10.1186/s41235-024-00586-2 (PMC11345344; doi:10.1186/s41235-024-00586-2)
Supplement: Supplementary file 1 [file 41235_2024_586_MOESM1_ESM.docx]

Effects of Machine Learning Errors on Human Decision Making: Manipulations of Model Accuracy, Error Types, and Error Importance

Supplemental Materials

Laura E. Matzen, Zoe N. Gastelum, Breannan C. Howell, Kristin M. Divis & Mallory C. Stites

Sandia National Laboratories

Correspondence should be addressed to Laura Matzen, P.O. Box 5800, Mail Stop 1327, Albuquerque, NM 87185-1327, E-mail: lematze@sandia.gov

In this document, we present supplemental information and analyses for the five experiments in the main manuscript.

**Experiment 1**

***Participants’ Estimates of Target Prevalence***

At the end of Experiment 1, the participants were asked to estimate what percentage of the trials contained targets. The results are shown in Figure 1. Only four participants guessed the correct target prevalence (60%). Nine participants overestimated the target prevalence, while 24 underestimated it. Since this was an unaided visual search task, it makes sense that many participants underestimated the target prevalence. The targets (Ts) were difficult to find without model assistance and the participants missed many of them.

*Figure 1.* A count of the number of participants giving each response to a question asking them to estimate how many trials in the experiment had contained targets in Experiment 1.

Figure 2 splits the Experiment 1 data into three groups: the participants who correctly estimated the target prevalence, the participants who overestimated the target prevalence, and the participants who underestimated the target prevalence. Although there are only a few participants in the first two groups, it is notable that the participants who estimated correctly had a relatively high average accuracy for the target present items compared to the other two groups. The participants who overestimated the target prevalence had relatively low accuracy for the target absent trials, indicating that they made several false alarms.

*Figure 2*. Average proportion correct for target present and target absent trials for participants who correctly estimated, overestimated, or underestimated the number of targets in Experiment 1.

**Experiment 2**

*Table 1.* The number of items representing each type of model output in the six error rate conditions.

| List | Hits | Correct Rejections | Misses | False Alarms | False Alarm/Misses |
| --- | --- | --- | --- | --- | --- |
| 50% Correct | 32 | 28 | 20 | 20 | 20 |
| 60% Correct | 40 | 32 | 16 | 16 | 16 |
| 70% Correct | 48 | 36 | 12 | 12 | 12 |
| 80% Correct | 56 | 40 | 8 | 8 | 8 |
| 90% Correct | 64 | 44 | 4 | 4 | 4 |
| 95% Correct | 68 | 46 | 2 | 2 | 2 |

***Analysis of Participants’ Overall Performance***

The participants’ average response times for the target absent and target present items from Experiment 2 are shown in Figure 3. Only correct responses were included in this analysis. As in Experiment 1, participants had slower response times for target absent items. However, as the model accuracy increased, the participants’ response times tended to decrease for both types of items.


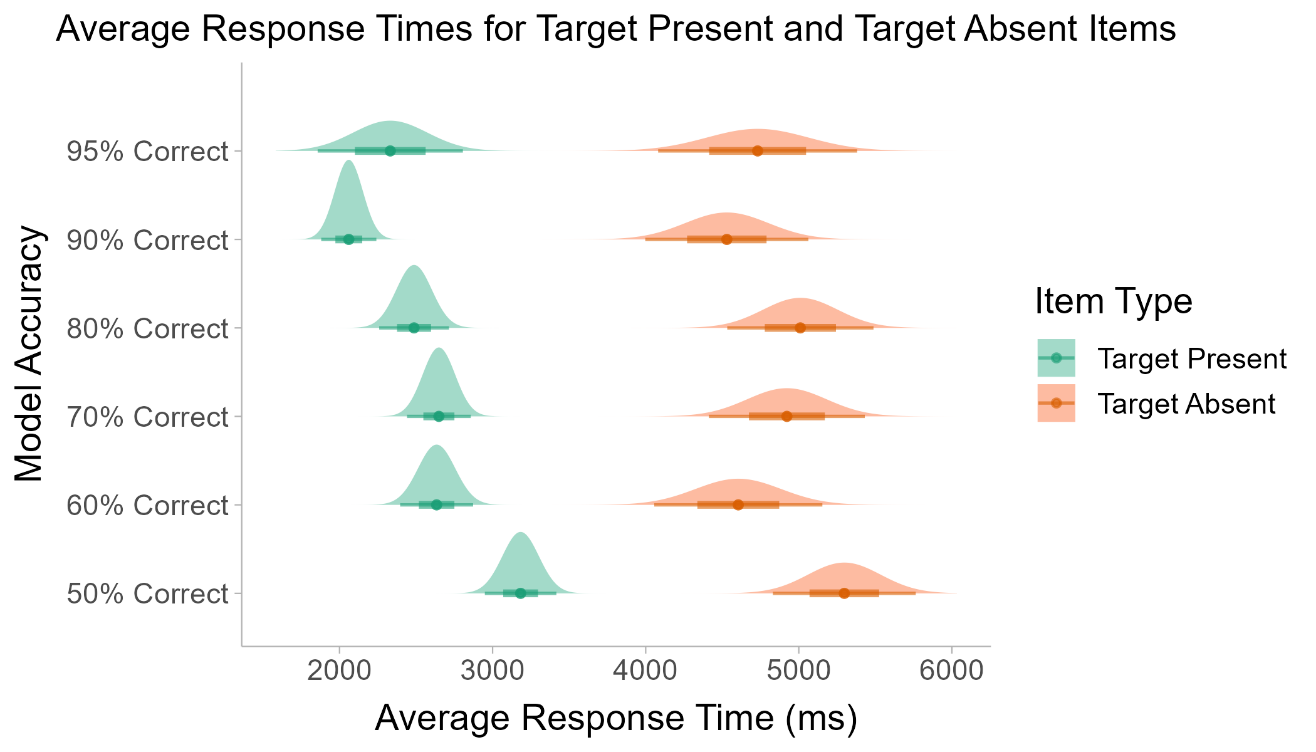


*Figure 3*. Average response times for correct responses to target absent and target present stimuli across all model accuracy conditions in Experiment 2.

**Experiment 3**

*Table 2.* The number of items of each type in each of the experimental lists in Experiment 3.

| List | Hits | Correct Rejections | Misses | False Alarms | False Alarm/Misses |
| --- | --- | --- | --- | --- | --- |
| 70% Correct,  All FAs | 72 | 12 | -- | 36 | -- |
| 70% Correct,  All Misses | 36 | 48 | 36 | -- | -- |
| 70% Correct,  All FA/misses | 36 | 48 | -- | -- | 36 |
| 70% Correct,  1/3 FAs | 48 | 36 | 24 | 12 | -- |
| 70% Correct,  1/2 FAs | 54 | 30 | 18 | 18 | -- |
| 70% Correct,  2/3 FAs | 60 | 24 | 12 | 24 | -- |
| 80% Correct,  All FAs | 72 | 24 | -- | 24 | -- |
| 80% Correct,  All Misses | 48 | 48 | 24 | -- | -- |
| 80% Correct,  All FA/misses | 48 | 48 | -- | -- | 24 |
| 80% Correct,  1/3 FAs | 56 | 40 | 16 | 8 | -- |
| 80% Correct,  1/2 FAs | 60 | 36 | 12 | 12 | -- |
| 80% Correct,  2/3 FAs | 64 | 32 | 8 | 16 | -- |
| 90% Correct,  All FAs | 72 | 36 | -- | 12 | -- |
| 90% Correct,  All Misses | 60 | 48 | 12 | -- | -- |
| 90% Correct,  All FA/misses | 60 | 48 | -- | -- | 12 |
| 90% Correct,  1/3 FAs | 64 | 44 | 8 | 4 | -- |
| 90% Correct,  1/2 FAs | 66 | 42 | 6 | 6 | -- |
| 90% Correct,  2/3 FAs | 68 | 40 | 4 | 8 | -- |

***Overall Accuracy for Single Error Type Lists***

Our first analysis assessed the differences in the participants’ overall accuracy for the nine lists in which all of the model errors within the list were of the same type (all FAs, all misses, or all FA/misses). The results of this analysis are shown in Figure 4. A 3x3 ANOVA (model accuracy by error type) showed that there was a significant main effect of model accuracy (*F*(2, 293) = 11.05, *p* < 0.001) and a significant main effect of error type (*F*(2, 293) = 8.11, *p* < 0.001), but there was not a significant interaction between the two (*F*(4, 293) = 1.98, *p* = 0.09).

Bonferroni pairwise comparisons showed that the participants’ overall accuracy was significantly lower when the model’s outputs were 70% correct than when they were 80% (*p* < 0.03) or 90% correct (*p* < 0.001). There was no significant difference in the participants’ overall accuracy when comparing lists where the model was 80% or 90% correct (*p* = 0.12). For the different error types, the participants’ overall accuracy was significantly lower when all of the model errors were misses than when they were FAs (*p* < 0.01) or FA/misses (*p* < 0.01). There was no significant difference between lists where all of the errors were FAs and lists where all of the errors were FA/misses (*t*(199) = 0.60).


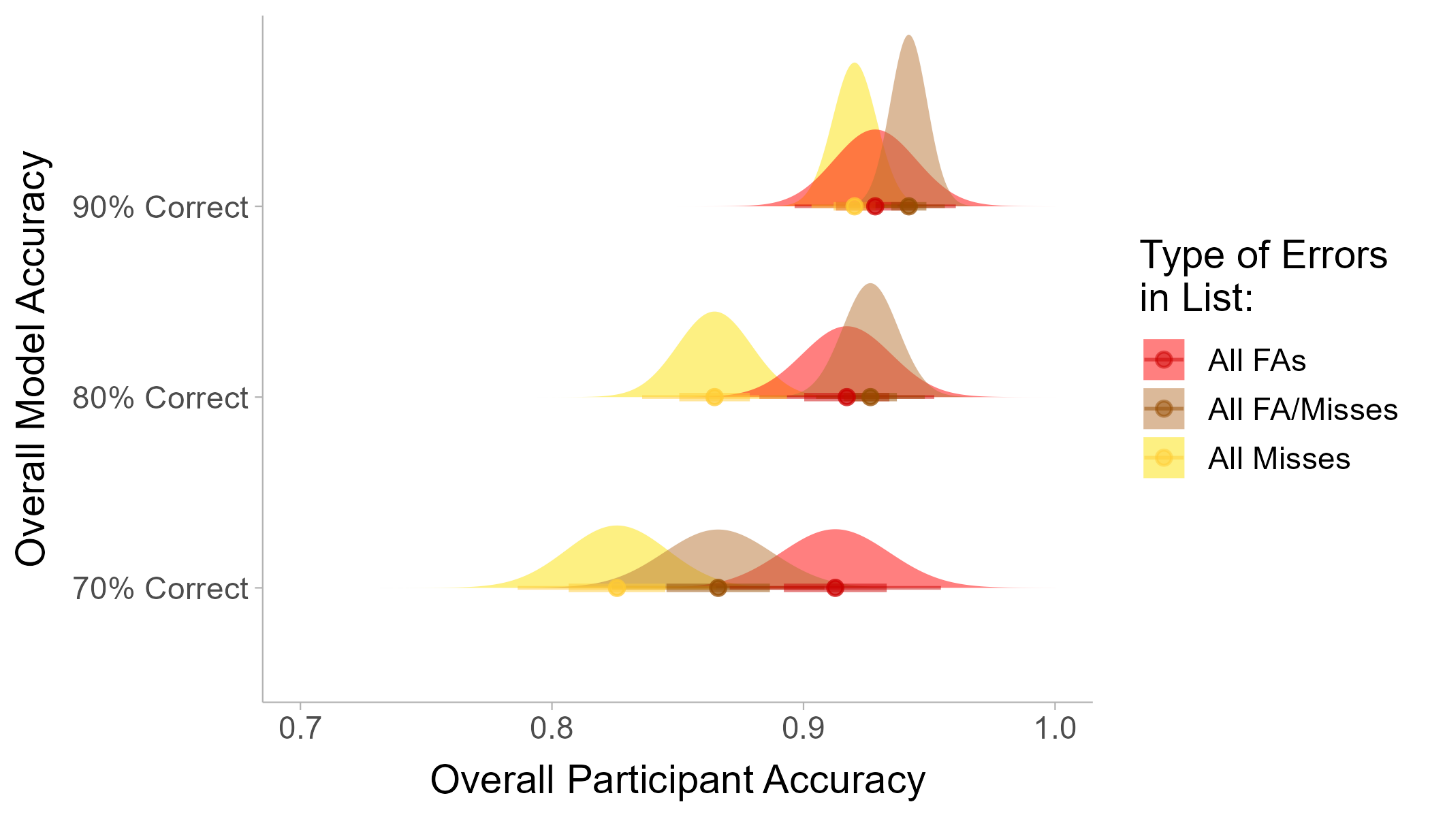


*Figure 4*. The participants’ overall accuracy for lists with only one type of error in Experiment 3.

***Overall Average Response Times for Single Error Type Lists***

Figure 5 shows the participants’ overall mean response times for correct responses on the nine lists in which all of the errors were of a single type. A 3x3 ANOVA (model accuracy by error type) showed that there was not a significant main effect of model accuracy (*F*(2, 293) = 1.81, *p* = 0.17), but there was a significant main effect of error type (*F*(2, 293) = 3.24, *p* < 0.05). There was not a significant interaction between model accuracy and error type (*F*(4, 293) = 0.63). Bonferroni pairwise comparisons showed that the participants’ average RT was significantly higher in the FA/miss conditions than in the FA conditions (*p* < 0.05). There were no other significant differences between conditions.

***
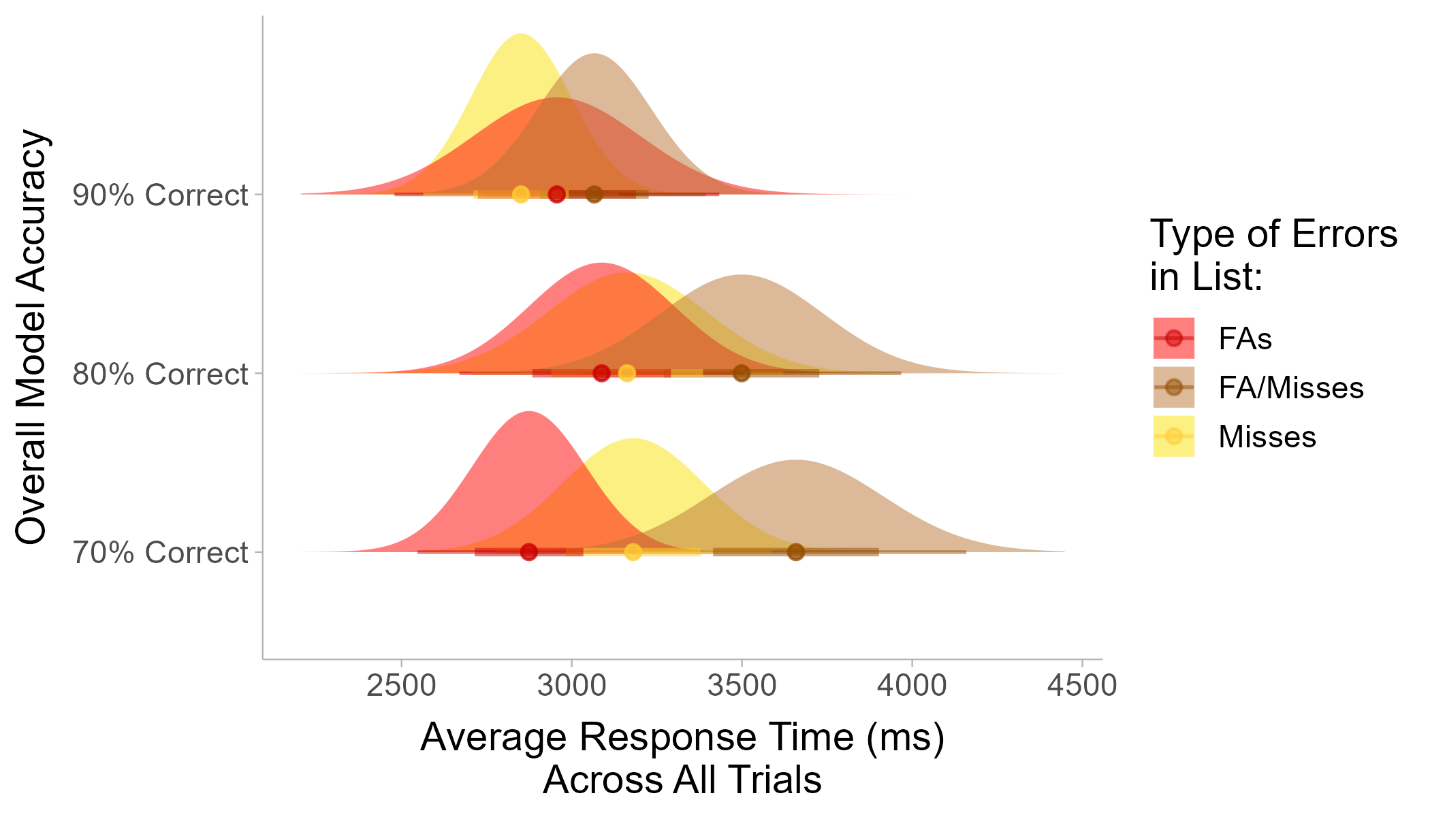
***

*Figure 5*. The participants’ average response times for all trials with correct responses in experiment conditions with only one type of model error in Experiment 3.

***Overall Accuracy and Response Times for Lists with Varying Proportions of FAs and Misses***

For our second analysis, we compared the lists that manipulated the proportion of FAs and misses in order to determine how the changing proportions of those two error types impacted the participants’ performance. Since the miss trials were consistently more difficult for participants, we expected that participants would have higher performance when there was a higher proportion of FAs among the model errors.

A 3x3 ANOVA (overall model accuracy by proportion of FAs) showed that there was a significant main effect of overall model accuracy (*F*(2, 276) = 20.37, *p* < 0.001) but there was not a significant main effect of the proportion of FAs (*F*(2, 276) = 0.52) nor was there a significant interaction (*F*(4, 276) = 1.35). Bonferroni pairwise comparisons showed that the participants’ overall accuracy was significantly lower when the model was 70% correct than when it was 80% (*p* < 0.01) or 90% correct (*p* < 0.001). Their accuracy was also significantly lower for the 80% correct model than for the 90% correct model (*p* < 0.001). These results are shown in Figure 6. A similar 3x3 ANOVA assessing the participants’ response times for correct trials did not find any significant differences between conditions (all *F*s < 2.09, all *p*s > 0.08).


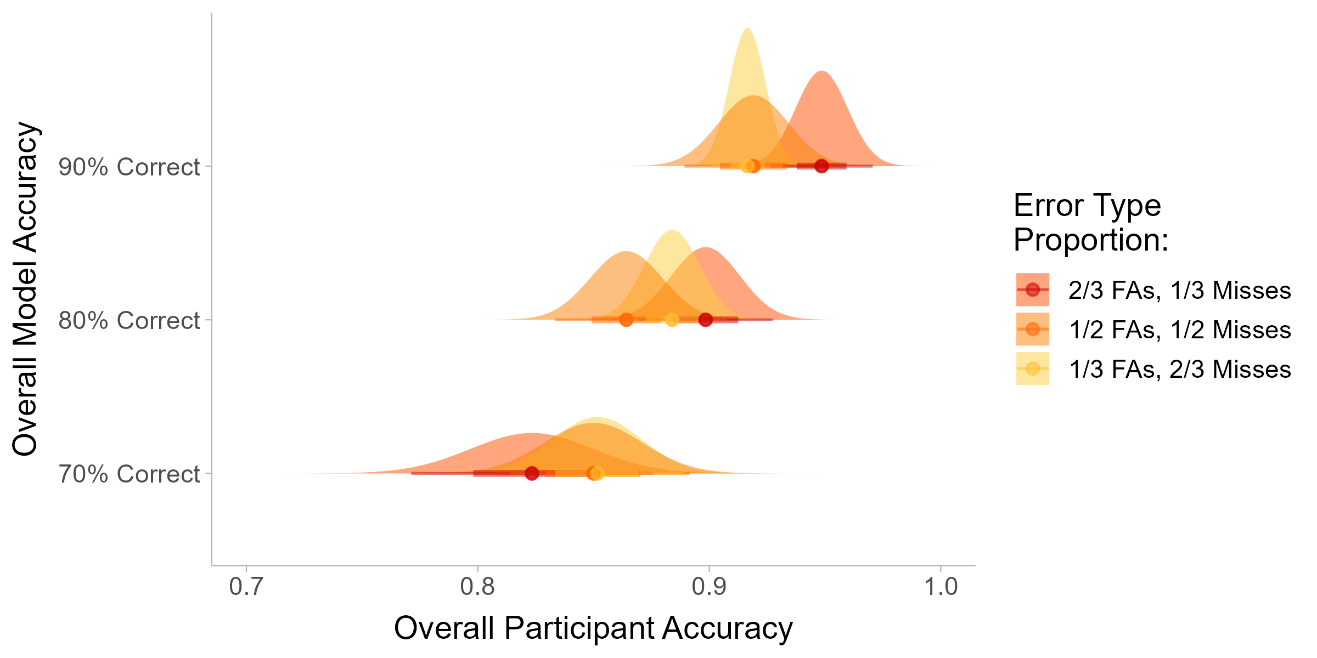


*Figure 6*. The participants’ average overall accuracy for the lists containing different proportions of ML false alarms and misses in Experiment 3.

**Experiment 4**

***Analysis of Participants’ Overall Performance***

A 3x3x2 ANOVA with overall model accuracy (70%, 80% or 90% correct overall) and item type emphasis (greater importance given to target present items, target absent items, or neither) as between-subjects factors and item type (target present or target absent) as a within-subjects factor found a significant main effect of overall model accuracy on the participants’ overall accuracy (*F*(2, 294) = 22.46, *p* < 0.001). There were no other significant main effects or interactions. Pairwise tests with Bonferroni correction showed that the participants’ accuracy was significantly higher in the 90% model accuracy condition relative to the 70% (*p* < 0.001) and 80% (*p* < 0.001) model accuracy conditions. The data are shown in Figure 8.


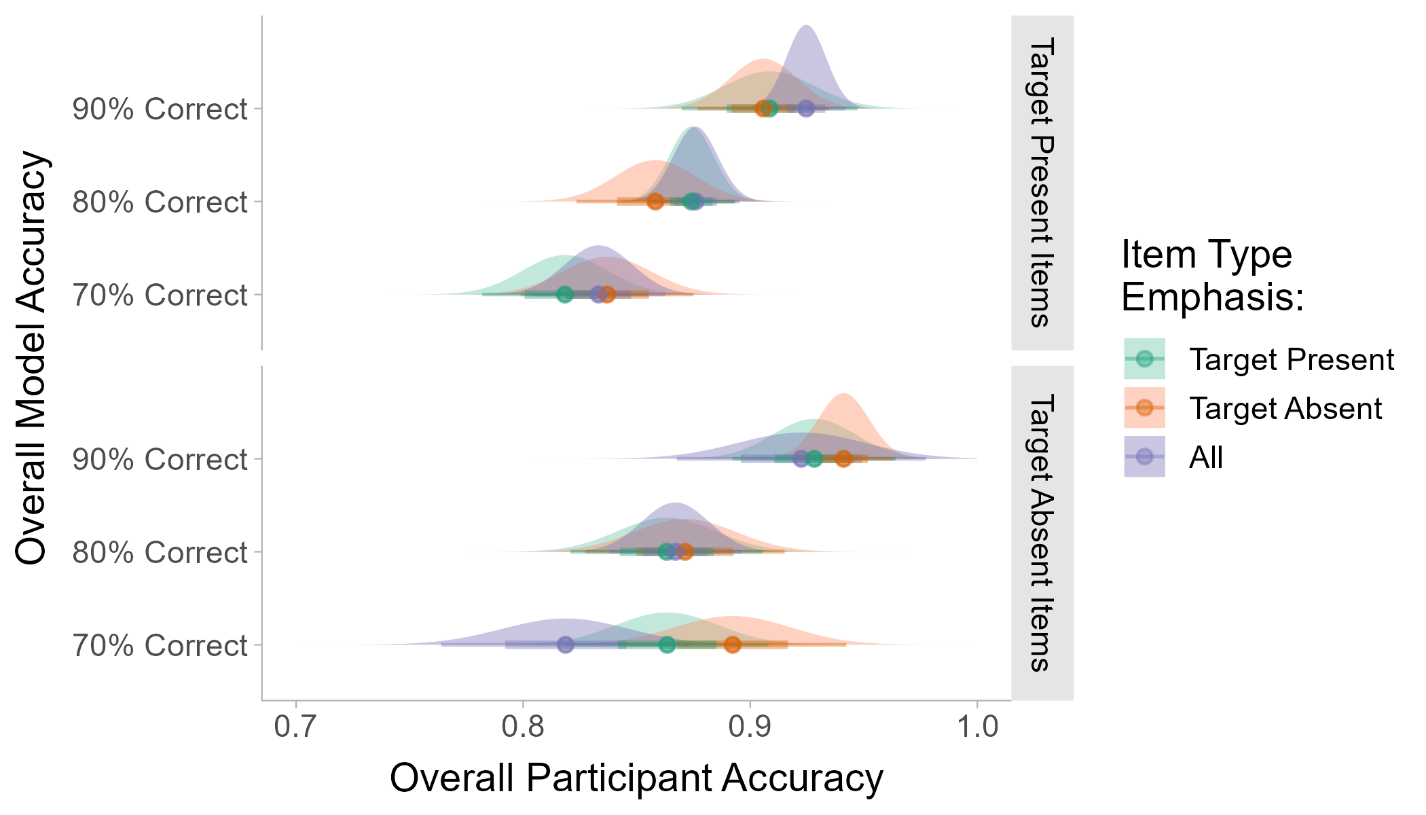


*Figure 8.* The participants’ average overall accuracy for all trials in each experimental list in Experiment 4.

A similar ANOVA for the RTs to correct trials found a significant main effect of item type emphasis (*F*(2, 294) = 3.83, *p* < 0.03) and a significant main effect of item type (*F*(1, 294) = 365.02, *p* < 0.001). There were no other significant main effects or interactions. Pairwise t-tests with Bonferroni correction showed that participants had significantly longer response times when the target absent items were emphasized relative to the condition where participants were just asked to do a good job overall (*p* < 0.01). They also had significantly slower RTs for the target absent images relative to the target present images (*p* < 0.001). These results are shown in Figure 9.


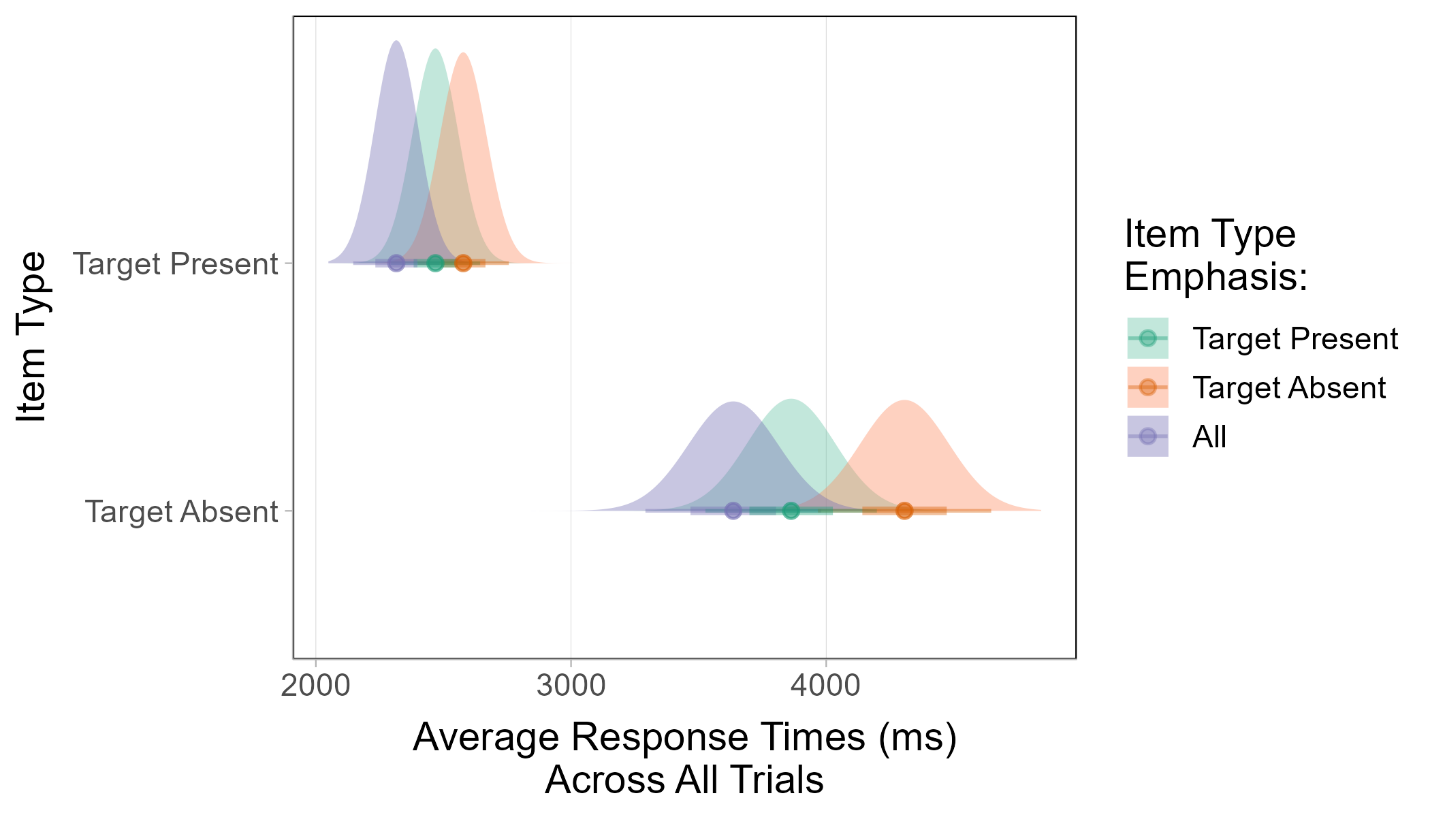


*Figure 9.* The participants’ average response times for all trials with correct responses to target present and target absent items in Experiment 4.

These results indicated that the participants had longer average response times for target absent trials when that type of item was deemed the most important. They spent more time on items without bounding boxes (correct rejection and miss trials) relative to other conditions, but this additional time did not translate to higher accuracy for those trials. We did not see a corresponding RT increase for target present trials when those types of trials were emphasized.

The participants’ average RTs for the trials containing model errors are shown in Figure 10. For the FA and FA/miss items, there were no significant main effects of interactions. As in the prior experiments, the participants had longer response times to the FA trials when they answered correctly, indicating that they were taking the time to check the contents of the bounding box. For the FA/miss trials, the participants had longer RTs when they answered incorrectly. This indicated that they realized that the bounding box did not contain a target and searched for another target in the image, but did not find the target. For the misses, there was a significant main effect of item type emphasis for the incorrect trials, as discussed in the main manuscript.


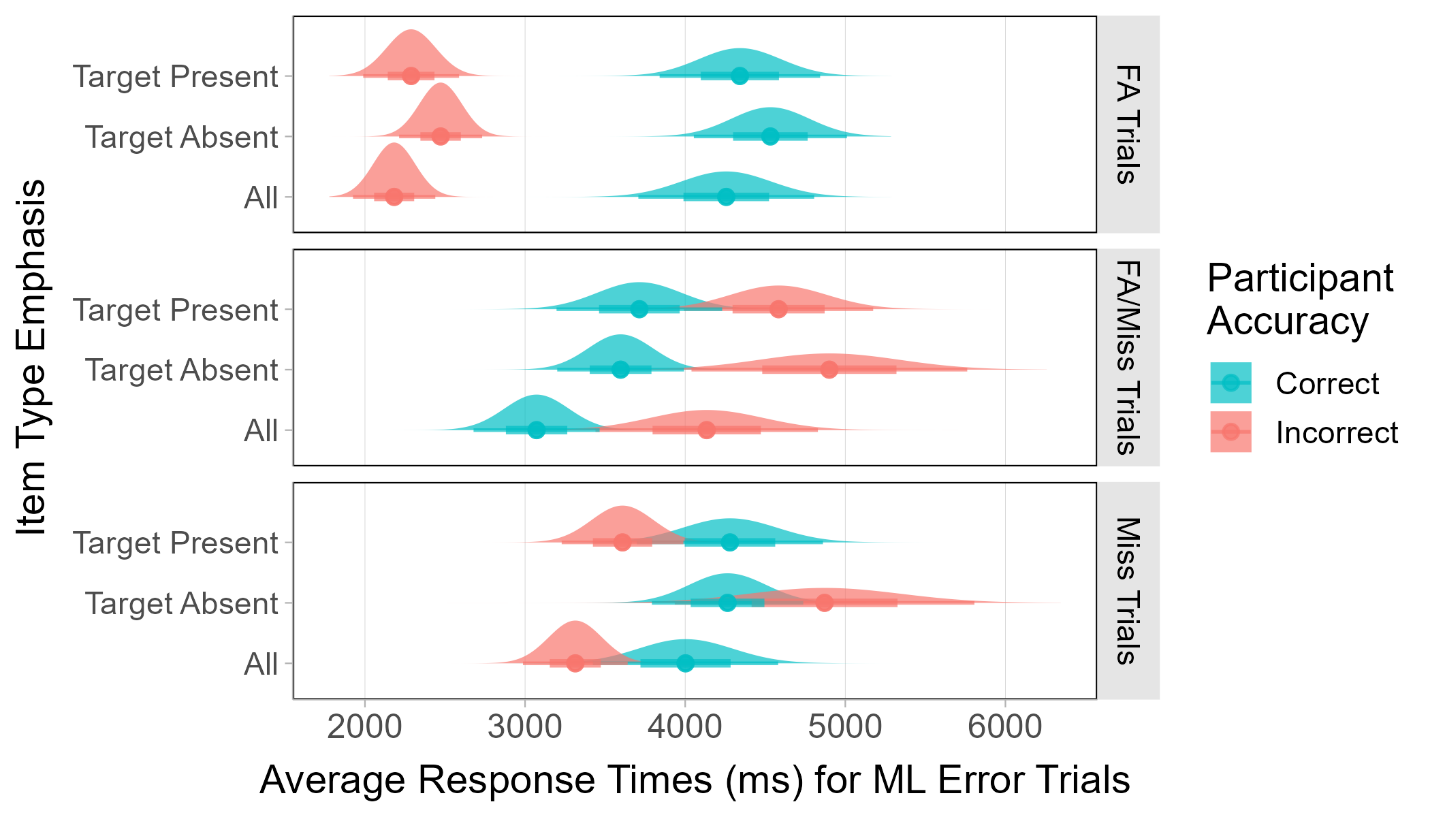


*Figure 10.* The participants’ average response times for correct and incorrect responses to model error trials under different conditions in Experiment 4.

**Experiment 5**

A 3x3x2 ANOVA with overall model accuracy (70%, 80% or 90% correct overall) and item type emphasis (greater importance given to target present items, target absent items, or neither) as between-subjects factors and item type (target present or target absent) as a within-subjects factor found a significant main effect of overall model accuracy on the participants’ overall accuracy (*F*(2, 291) = 42.92, *p* < 0.001). Pairwise tests with Bonferroni correction showed that the participants’ accuracy was significantly different across all levels of overall model accuracy (all *p*s < 0.001). There was also a significant main effect of item type (*F*(1, 219) = 4.14, *p* < 0.05). Overall, the participants had higher accuracy for target absent items than for target present items. There was not a significant main effect of error emphasis, nor any significant interactions. These results are shown in Figure 11.


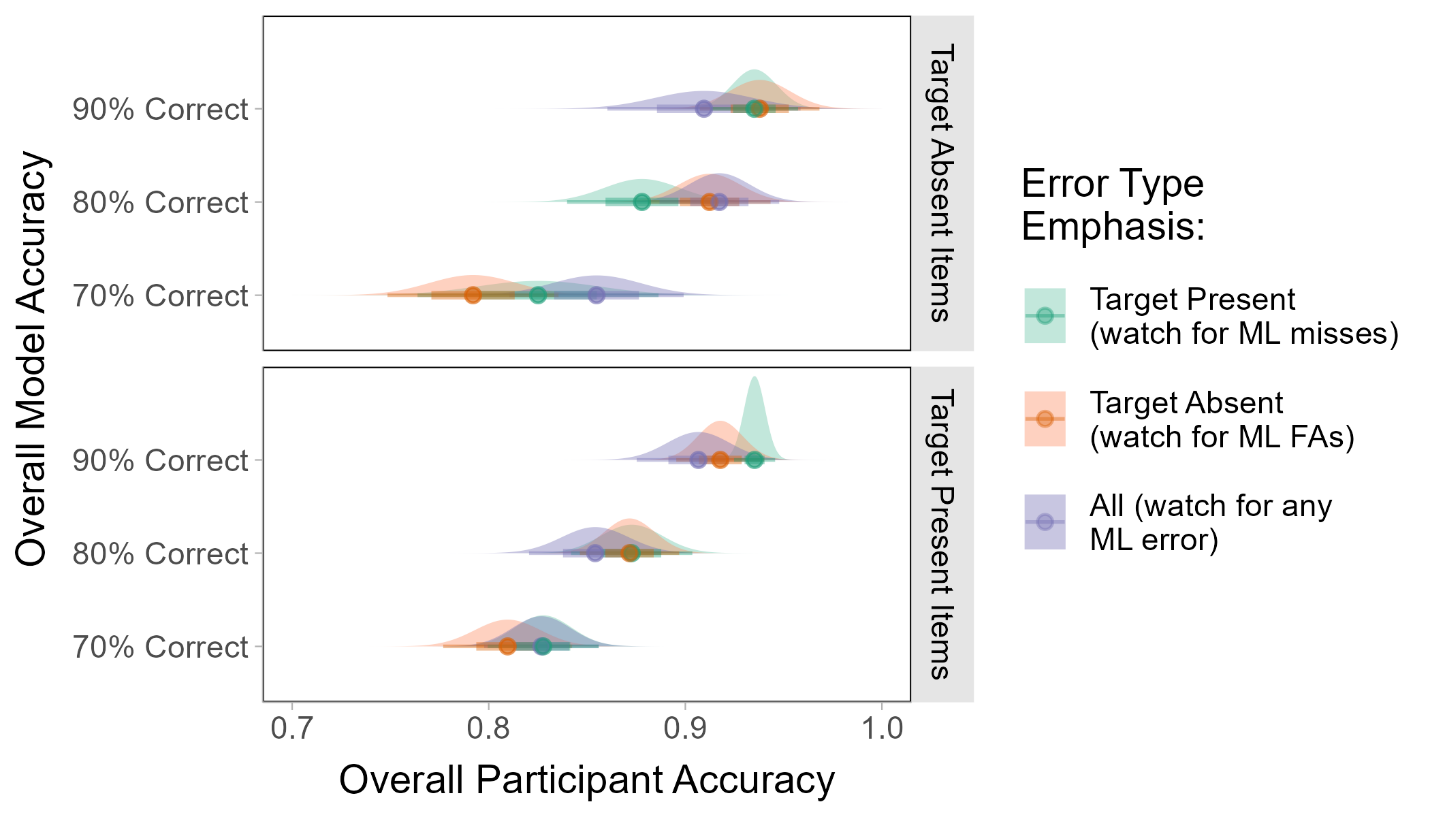


Figure 11. The participants’ average overall accuracy for target present and target absent items in conditions emphasizing different types of ML errors in Experiment 5..

A similar ANOVA for the RTs on trials where participants answered correctly found a significant main effect of item type (*F*(1, 291) = 307.64, *p* < 0.001). The participants had significantly longer RTs for target absent trials than for target present trials (*p* < 0.001). There were no significant main effects or interactions (all *F*s < 2.87, all ps > 0.05). The participants’ average response times to target present and target absent items (collapsed across overall model accuracy) are shown in Figure 12.


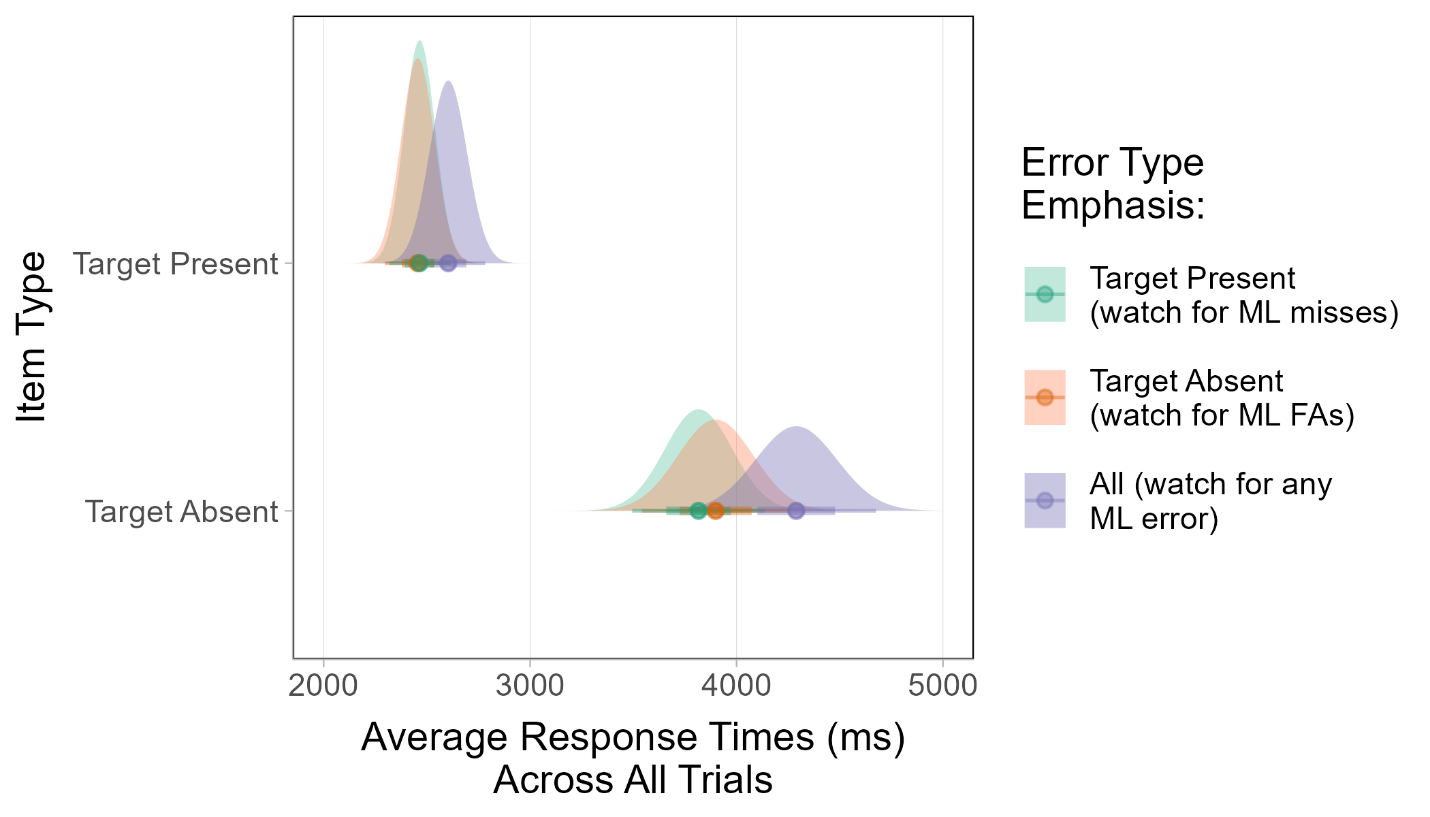


Figure 12. The participants’ average response times for correct responses to target present and target absent items in Experiment 5.

The participants’ average RTs for the model error trials are shown in Figure 13. The results for the FA and FA/miss trials mirrored the results of Experiment 4. Unlike Experiment 4, there was no significant main effect for the trials where the model missed a target. In this case, the participants consistently had longer RTs when they responded correctly (i.e., when they found the missing target) than when they didn’t.


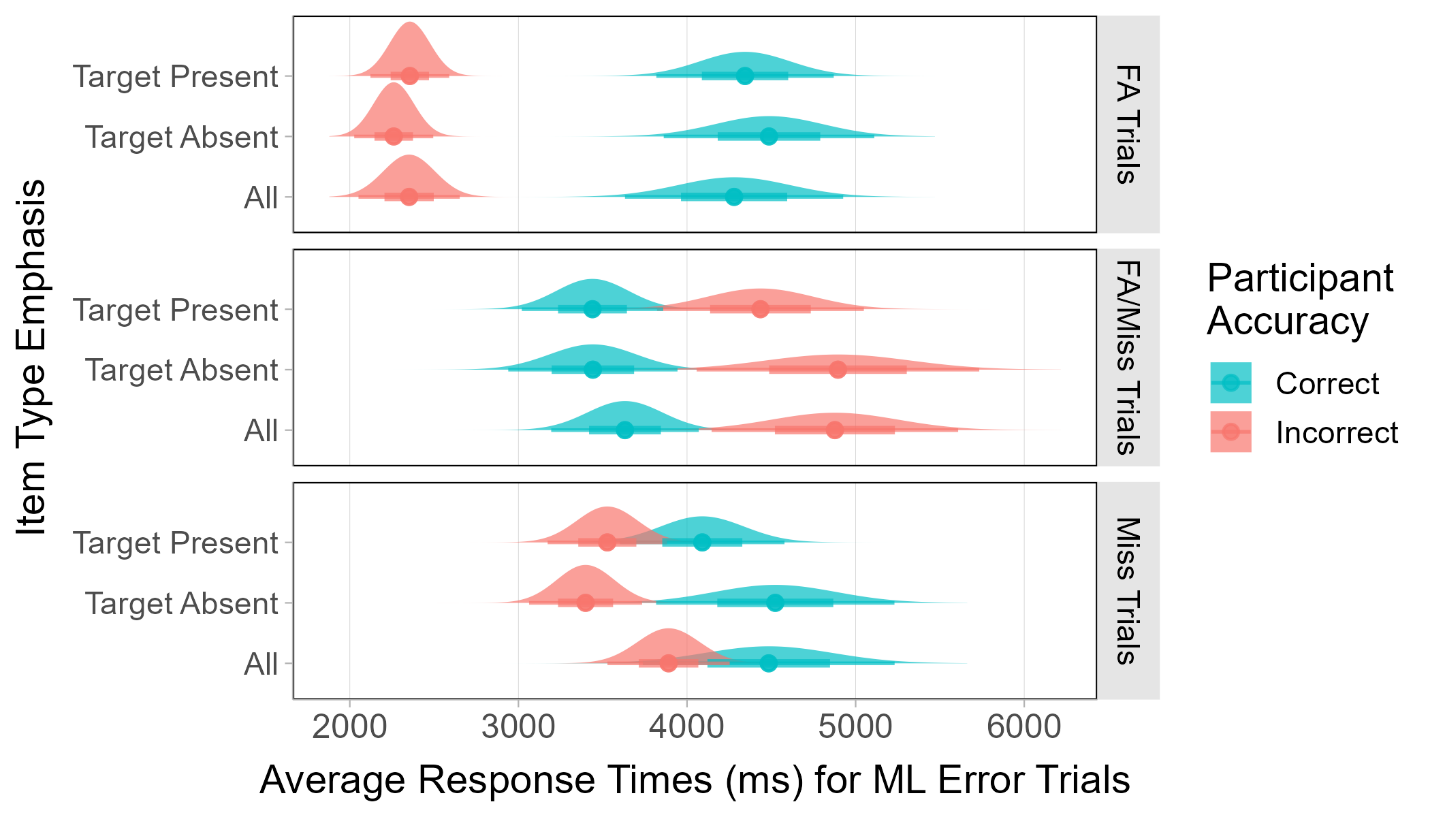


*Figure 13.* The participants’ average response times for correct and incorrect responses to model error trials in Experiment 5.

**Signal Detection Theory Analysis**

The five experiments in this study varied the number of targets that were correctly detected by the mock ML outputs, as well as the types of errors that were made and the relative importance of different types of stimuli or different types of errors. Each of these factors could change the participants’ decision criterion and their sensitivity (their ability to distinguish between target present and target absent stimuli). Prior studies (cf. Drew, Guthrie & Reback, 2020; Kunar, 2022) have used signal detection theory to assess how computer aided detection affects *d’* (a measure of sensitivity) and *c* (a measure of criterion). Table 3 shows the *d’* values and Table 4 shows the *c* values for each condition in each experiment. Note that in these experiments, the participants’ false alarm rates were very low, and numerous participants never made a false alarm. To calculate the signal detection measures for participants with zero false alarms, the false alarm rate was corrected to 1/(2n) where n was the number of Target Absent trials. A similar correction was applied when participants had a perfect hit rate.

*Table 3.* The participants’ average *d’* scores (and standard deviation) for all experiments and conditions.

|  | | **No ML outputs** | **Mock ML outputs with an overall accuracy of:** | | | | | |
| --- | --- | --- | --- | --- | --- | --- | --- | --- |
|  |  |  | **50%** | **60%** | **70%** | **80%** | **90%** | **95%** |
| **Expt 1** | Baseline | 2.36 (0.88) |  |  |  |  |  |  |
| **Expt 2** | ⅓ of errors are FAs, ⅓ are misses, ⅓ are FA/misses |  | 3.10 (0.50) | 3.10 (0.75) | 3.34 (0.74) | 3.47  (0.57) | 3.81  (0.49) | 4.03 (0.52) |
| **Expt 3** | All errors are FAs |  |  |  | 3.51 (1.21) | 3.48 (1.25) | 3.59 (1.20) |  |
|  | All errors are misses |  |  |  | 2.75 (0.79) | 2.98 (0.71) | 3.36 (0.63) |  |
|  | All errors are FA/misses |  |  |  | 3.24 (0.87) | 3.53 (0.78) | 3.60 (0.67) |  |
|  | ⅔ of errors are FAs, ⅓ are misses |  |  |  | 2.34 (1.48) | 2.93 (1.00) | 3.75 (0.73) |  |
|  | ½ of errors are FAs, ½ are misses |  |  |  | 2.54 (1.19) | 2.56 (1.01) | 3.18 (1.01) |  |
|  | ⅓ of errors are FAs, ⅔ are misses |  |  |  | 2.65 (1.12) | 2.82 (0.83) | 3.06 (0.73) |  |
| **Expt 4** | All items important |  |  |  | 2.23 (1.00) | 2.47 (0.79) | 3.21 (0.82) |  |
|  | Target Present items most important |  |  |  | 2.37 (0.90) | 2.54 (0.91) | 3.19 (0.89) |  |
|  | Target Absent items most important |  |  |  | 2.67 (1.04) | 2.54 (0.98) | 3.16 (0.78) |  |
| **Expt 5** | All model errors are important |  |  |  | 2.31 (0.94) | 2.77 (0.84) | 3.11 (0.88) |  |
|  | Model FAs most important |  |  |  | 1.90 (0.88) | 2.86 (0.95) | 3.25 (0.85) |  |
|  | Model misses most important |  |  |  | 2.22 (1.01) | 2.60 (0.79) | 3.24 (0.66) |  |

*Table 4.* The participants’ average *c* values (and standard deviation) for all experiments and conditions.

|  | | **No ML outputs** | **Mock ML outputs with an overall accuracy of:** | | | | | |
| --- | --- | --- | --- | --- | --- | --- | --- | --- |
|  |  |  | **50%** | **60%** | **70%** | **80%** | **90%** | **95%** |
| **Expt 1** | Baseline | 0.51 (0.54) |  |  |  |  |  |  |
| **Expt 2** | ⅓ of errors are FAs, ⅓ are misses, ⅓ are FA/misses |  | 0.65 (0.25) | 0.51 (0.31) | 0.41 (0.28) | 0.37  (0.25) | 0.20  (0.21) | 0.07 (0.24) |
| **Expt 3** | All errors are FAs |  |  |  | -0.29 (0.63) | -0.35 (0.47) | -0.29 (0.35) |  |
|  | All errors are misses |  |  |  | 0.64 (0.38) | 0.61 (0.23) | 0.39 (0.26) |  |
|  | All errors are FA/misses |  |  |  | 0.60 (0.40) | 0.33 (0.30) | 0.26 (0.28) |  |
|  | ⅔ of errors are FAs, ⅓ are misses |  |  |  | -0.13 (0.54) | 0.11 (0.27) | 0.17 (0.32) |  |
|  | ½ of errors are FAs, ½ are misses |  |  |  | 0.19 (0.30) | 0.15 (0.29) | 0.02 (0.37) |  |
|  | ⅓ of errors are FAs, ⅔ are misses |  |  |  | 0.34 (0.25) | 0.31 (0.21) | 0.18 (0.18) |  |
| **Expt 4** | All items important |  |  |  | 0.07 (0.42) | 0.03 (0.26) | 0.10 (0.37) |  |
|  | Target Present items most important |  |  |  | 0.21 (0.44) | 0.05 (0.33) | 0.11 (0.33) |  |
|  | Target Absent items most important |  |  |  | 0.26 (0.38) | 0.10 (0.33) | 0.15 (0.26) |  |
| **Expt 5** | All model errors are important |  |  |  | 0.17 (0.34) | 0.25 (0.35) | 0.09 (0.44) |  |
|  | Model FAs most important |  |  |  | 0.02 (0.30) | 0.19 (0.33) | 0.14 (0.25) |  |
|  | Model misses most important |  |  |  | 0.12 (0.39) | 0.10 (0.39) | 0.05 (0.24) |  |

For each experiment, ANOVAs were used to analyze whether *d’* and *c* differed across conditions.

**Experiment 2**

In Experiment 2, a one-way ANOVA showed that changing the ML error rate had a significant impact on *d’* (*F*(5, 202) = 13.41, *p* < 0.001). Pairwise t-tests with Bonferroni correction showed that *d’* was significantly higher in the 90% and 95% correct conditions than they were in the 50%, 60%, or 70% correct conditions (all *p*s < 0.001). There was also a significant difference between the 80% and 95% correct conditions (*p* < 0.01).

The manipulation of the ML error rate also had a significant impact on *c* (*F*(5, 202) = 22.84, *p* < 0.001). The participants’ criterion decreased as the overall model accuracy increased, indicating that they were more likely to respond “target present” when the model accuracy was higher. This difference was significant when comparing the 50% correct condition to all conditions (all *p*s < 0.01) except for the 60% correct condition (*p* = 0.37), when comparing the 60% correct condition to the 90% and 95% correct conditions (both *p*s < 0.001), and when comparing the 70% and 80% correct conditions to the 95% correct condition (both ps < 0.001).

**Experiment 3**

In Experiment 3, the lists with one type of model error and the lists with different proportions of FAs and misses were analyzed separately.

***Lists with a Single Error Type***

For the lists with one type of model error, a two-way ANOVA found a significant main effect of overall model accuracy (*F*(2, 293) = 3.39, *p* < 0.05), and a significant main effect of error type (*F*(2, 293) = 8.40, *p* < 0.001) on *d’*. There was not a significant interaction (*F*(4, 293) = 0.80, *p* = 0.53). Pairwise t-tests with Bonferroni correction showed that *d’* was significantly higher in the 90% correct condition than in the 70% correct condition (*p* < 0.01). For the error type conditions, *d’* was significantly lower when all the errors were misses than when all the errors were FAs (*p* < 0.001) or when all the errors were FA/Misses (*p* < 0.01).

A similar analysis of *c* found a significant main effect of overall model accuracy (*F*(2, 293) = 6.62, *p* < 0.01), a significant main effect of error type (*F*(2, 293) = 145.29, *p* < 0.001), and a significant interaction between the two (*F*(4, 293) = 2.47, *p* < 0.05). Pairwise t-tests with Bonferroni correction showed that for the conditions where all the model errors were FAs, there were no significant differences in *c* for the different error rate conditions. The value of *c* was negative in all of these conditions, indicating a bias towards a more liberal criterion (more “target present” responses). For the conditions where all the model errors were misses, *c* was significantly lower in the 90% correct condition than in the 70% and 80% correct conditions (both *p*s < 0.01). This indicates that the participants were more likely to respond “target present” when the model missed fewer targets. When all the model errors were FA/misses, *c* was significantly higher in the 70% correct condition than in the 80% or 90% correct conditions (both *p*s < 0.01). Once again, the participants were more likely to respond “target present” when the model accuracy was higher. This provides evidence that the participants were correctly recognizing that the model had produced a false alarm, but then they failed to find the true targets in the model error trials. Comparing the different model error conditions to one another showed that *c* was significantly lower for the all-FA lists than for the all-miss or all-FA/miss lists across all levels of model accuracy (all *p*s < 0.001). This shows that participants had the strongest bias towards responding “target present” in the all-FA conditions.

***Lists with Different Proportions of Misses and False Alarms***

For the lists with different proportions of misses and FAs, an ANOVA found a significant main effect of overall model accuracy on *d’* (*F*(2, 276) = 15.66, *p* < 0.001). There was not a significant main effect of the proportion of FAs (*F*(2, 276) = 1.40, *p* = 0.25), nor was there a significant interaction (*F*(4, 276) = 2.24, *p* = 0.07). Pairwise t-tests with Bonferroni correction showed that *d’* was significantly higher in the 90% correct condition than in the 70% or 80% correct conditions (both *p*s < 0.001).

A similar analysis of *c* found a significant main effect of the proportion of FAs (*F*(2, 276) = 12.98, *p* < 0.001) and a significant interaction between the proportion of FAs and the overall model accuracy (*F*(4, 276) = 5.62, *p* < 0.001), but there was not a significant main effect of overall accuracy (*F*(2, 276) = 1.11, *p* = 0.33). Pairwise t-tests with Bonferroni correction showed that when the model had an overall accuracy of 70%, *c* was significantly lower in the condition where ⅔ of the model errors were FAs than in the conditions that had lower proportions of FAs and higher proportions of misses (both *p*s < 0.01). This indicates that the participants were more likely to respond “target present” when there was a higher proportion of FAs. When the model was 80% correct, *c* was significantly lower in the conditions where ⅔ or ½ of the model errors were FAs than in the condition where only ⅓ of the model errors were FAs (both *p*s < 0.05). Once again, this shows that the participants were more likely to respond “target present” when there were more FAs. When the model was 90% correct overall, there were no significant differences between the conditions with different proportions of FAs (all *p*s > 0.12).

**Experiment 4**

The analysis of the *d’* values in Experiment 4 found a significant main effect of overall model accuracy (*F*(2, 294) = 21.50, *p* < 0.001), but no main effect of the item type emphasis manipulation (*F*(2, 294) = 0.73, *p* = 0.48) and no interaction (*F*(4, 294) = 0.68, *p* = 0.64). Pairwise t-tests with Bonferroni correction showed that, once again, *d’* was significantly higher when the model was 90% correct than when it was 70% or 80% correct (both *p*s < 0.001).

An analysis of *c* did not find significant main effects of overall model accuracy (*F*(2, 294) = 2.82, *p* = 0.06) or item type emphasis (g(2, 294) = 2.28, *p* = 0.10). There was not a significant interaction between the two factors (*F*(4, 294) = 0.47, *p* = 0.76).

**Experiment 5**

The results for Experiment 5 were very similar to the results of Experiment 4. The analysis of *d’* found a significant main effect of overall model accuracy (*F*(2, 291) = 35.61, *p* < 0.001), but no main effect of the emphasis on one error type over another (*F*(2, 291) = 0.14, *p* = 0.87) and no interaction (*F*(4, 291) = 1.44, *p* = 0.22). The analysis of c found no significant main effects (overall model accuracy: *F*(2, 291) = 1.87, *p* = 0.16; error type emphasis: *F*(2, 291) = 1.34, *p* = 0.26)) and no interaction (*F*(4, 291) = 1.08, *p* = 0.36).

**Discussion of Signal Detection Analysis**

The analyses of *d’* and *c* showed that changing the rate of model errors and the types of errors produced by the model changed the participants’ sensitivity and shifted their decision criteria. Unsurprisingly, when the model outputs were more accurate overall, the participants’ *d’* scores increased. This analysis also supported the finding in the main paper that the participants were better at detecting the model’s FAs than they were at detecting the model’s misses. Their *d’* scores were higher when the model made fewer misses.

The analysis of *c* showed that participants were more likely to respond “target present” as the model accuracy increased and in conditions that had a higher proportion of FAs. The presence of more bounding boxes shifted the participants towards a more liberal criterion, even though some of the bounding boxes were inaccurate. This is consistent with our observation in Experiment 3 that a subset of the participants responded “target present” for *all* of the FA items, indicating that they were responding to the presence of a bounding box without checking it thoroughly. The number of participants who adopted this strategy increased as the model accuracy increased. Although only a subset of the participants adopted this all-or-nothing approach of responding “target present” to every stimulus that contained a bounding box, our analysis of *c* indicates a consistent shift toward greater complacency with the bounding boxes and a more liberal criterion when the model made fewer errors and when more of those errors were FAs.

In Experiments 4 and 5, we attempted to shift the criterion by changing the task instructions. Experiment 4 emphasized the importance of one type of item (target present or target absent) and Experiment 5 emphasized the importance of detecting FAs or misses produced by the model. In Experiment 4, our analysis of the ML error trials showed that the participants in the condition that emphasized target absent trials spent more time searching the images without a bounding box and were more likely to find the missed targets. The signal detection analysis found that *c* was higher for the participants in this group, indicating that they tended to use a more conservative criterion. However, they did not differ significantly from the groups whose instructions emphasized target present items or all items equally. Similarly, for Experiment 5, we found that the emphasis on different types of ML errors did not have a significant effect on *d’* or *c*. Notably, these results are similar to those of Kunar & Watson (2023), who found that warning participants about errors in a computer aided detection system had little impact on their performance. It appears that warning people about errors in automated systems is not enough to shift their decision criterion unless they are told not to use the outputs at all (Kunar & Watson, 2023). As ML and AI-based systems proliferate, it is becoming increasingly important to help users to appropriately calibrate their usage and trust of AI/ML outputs. Additional research is needed to determine whether other methods of warning users about the potential for model errors will impact their sensitivity, criterion, and/or their trust in the automated system.
